# A novel long non-coding RNA-PRLB acts as a tumor promoter through regulating miR-4766-5p/SIRT1 axis in breast cancer

**DOI:** 10.1038/s41419-018-0582-1

**Published:** 2018-05-11

**Authors:** Yiran Liang, Xiaojin Song, Yaming Li, Yuting Sang, Ning Zhang, Hanwen Zhang, Ying Liu, Yi Duan, Bing Chen, Renbo Guo, Wenjing Zhao, Lijuan Wang, Qifeng Yang

**Affiliations:** 1 Department of Breast Surgery, Qilu Hospital, Shandong University, 250012 Jinan, Shandong China; 2 Pathology Tissue Bank, Qilu Hospital, Shandong University, 250012 Jinan, Shandong China; 3 Department of Urology, Shandong Cancer Hospital affiliated to Shandong University, 250117 Jinan, Shandong China

## Abstract

Accumulating evidence indicates that long non-coding RNAs (lncRNAs) play a critical role in cancerous processes as either oncogenes or tumor suppressor genes. Here, we demonstrated that lncRNA-PRLB (progression-associated lncRNA in breast cancer) was upregulated in human breast cancer tissues and breast cancer cell lines. Further evaluation verified that lncRNA-PRLB was positively correlated with the extent of metastasis, and its expression was correlated with shorter survival time of breast cancer patients. We identified microRNA miR-4766-5p as an inhibitory target of lncRNA-PRLB. Both lncRNA-PRLB overexpression and miR-4766-5p knockdown could remarkably enhance cell growth, metastasis, and chemoresistance. We also determined that sirtuin 1 (SIRT1) was an inhibitory target of miR-4766-5p, and that SIRT1 was inhibited by both lncRNA-PRLB knockdown and miR-4766-5p overexpression. Significantly, we found that the promotion of cell proliferation and metastasis, the acquisition of chemoresistance, and the increased expression of SIRT1 induced by lncRNA-PRLB overexpression could be partly abrogated by ectopic expression of miR-4766-5p. Taken together, our findings indicated that lncRNA could regulate the progression and chemoresistance of breast cancer via modulating the expression levels of miR-4766-5p and SIRT1, which may have a pivotal role in breast cancer treatment and prognosis prediction.

## Introduction

Breast cancer is one of the most common malignancy among women and the incidence increases gradually in the world^1^. Although the combination of surgery and adjuvant therapy can effectively improve the prognosis of patients with breast cancer, the occurrence of metastasis and chemoresistance could lead to disease recurrence and cancer-associated mortality^2, 3^. The development of breast cancer is a complicated process involving accumulation of both genetic and epigenetic changes. Therefore, further study of the precise molecular mechanisms involved in the progression and chemoresistance is essential for the reduction of mortality and easing the burden caused by breast cancer.

Recently, emerging evidence suggested that long non-coding RNAs (lncRNAs) played essential roles in human diseases, especially in tumors^4, 5^. LncRNAs are defined as a class of non-coding RNAs (ncRNAs) that are longer than 200 nucleotides^6^. Although lncRNAs lack cross-species conservation^7, 8^, increasing evidence suggests that lncRNAs play important roles in a variety of cellular process, and therefore may contribute to tumor initiation, metastasis, and chemoresistance^9–12^. LncRNA HOTAIR, which is one of the most famous lncRNAs, played significant role in lung cancer^13^, renal cancer^14^, esophageal cancer^15^, ovarian cancer^16^, and so on. LncRNA-MALAT1 promoted lung cancer metastasis and could serve as a treatment target^17^. Linc00152 was overexpressed in breast cancer and participated in regulating cell proliferation^18^. LncRNA CRNDE was originally found to be increased in colorectal cancer^19^ and could promote cell proliferation, metastasis, and chemoresistance^20, 21^. However, although a few lncRNAs have been reported, more studies were needed to clarify the regulation mechanism of lncRNAs in breast cancer.

Emerging evidences suggest that lncRNA may function as a competing endogenous RNA (ceRNA) in modulating the expression and biological functions of microRNA^22–24^. In the present study, we identified a novel lncRNA-PRLB (progression-associated lncRNA in breast cancer) as a tumor promoter in breast cancer. Furthermore, lncRNA-PRLB was associated with breast cancer progression and chemoresistance by regulating the expression of miR-4766-5p. Our findings suggested that the inhibition of lncRNA-PRLB represented a promising therapeutic strategy for breast cancer treatment.

## Results

### LncRNA-PRLB is upregulated in breast cancer and is associated with advanced tumor stage and poor prognosis

**Fig. 1:**
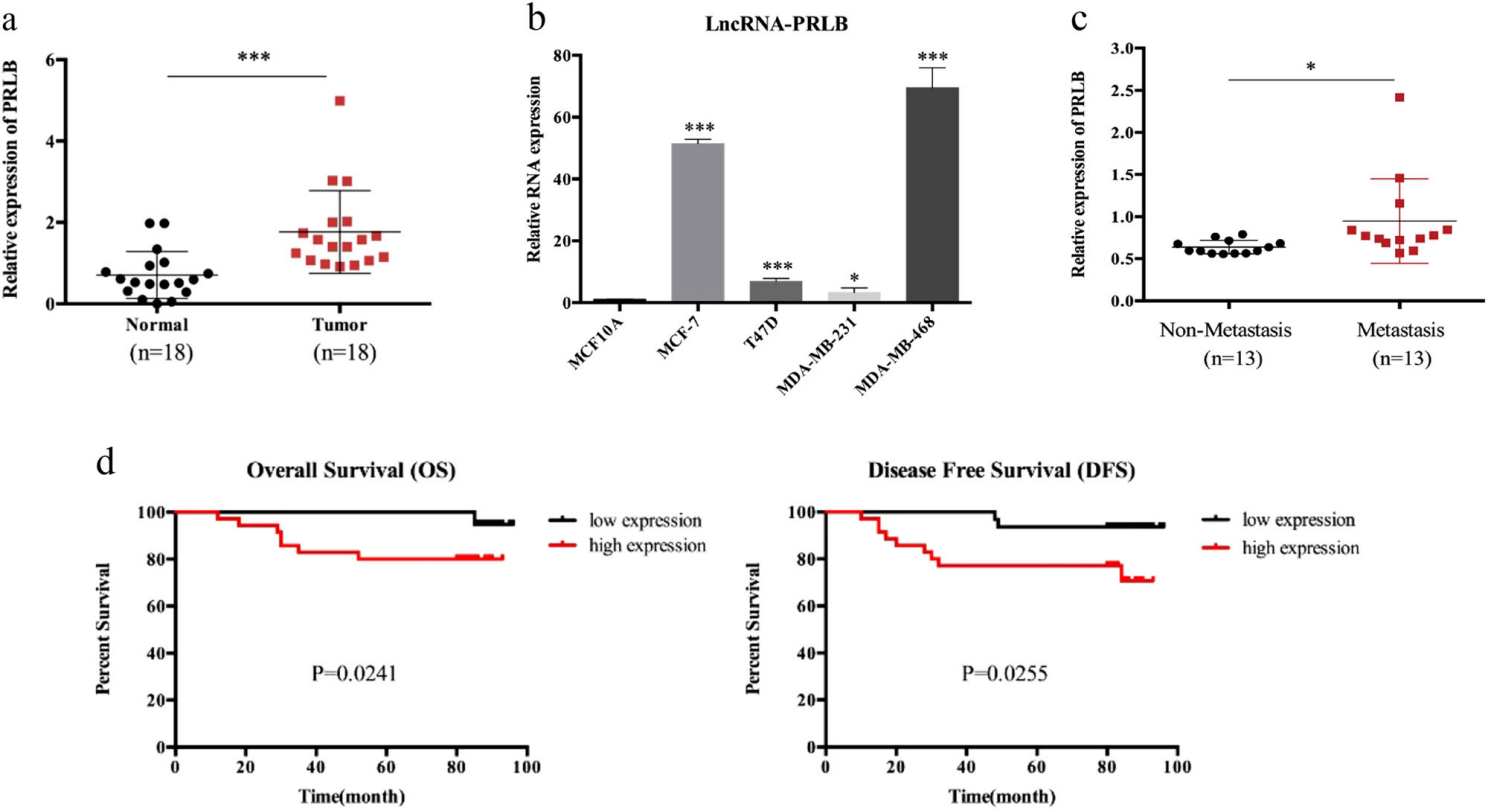
Relative lncRNA-PRLB expression and its clinical significance in breast cancer tissues. **a** The relative expression of lncRNA-PRLB in breast cancer tissues (*n* = 18) compared with corresponding non-tumor tissues (*n* = 18). LncRNA-PRLB expression was examined by qRT-PCR and normalized to actin expression. **b** LncRNA-PRLB expression was higher in breast cancer cell lines than in normal breast epithelial cell line MCF-10A. **c** Expression of lncRNA-PRLB in primary breast cancer with or without metastasis. **d** Kaplan–Meier analysis of overall survival and disease-free survival of 68 patients with breast cancer based on lncRNA-PRLB expression. *P < 0.05, **P < 0.01, and ***P < 0.001

To identify lncRNAs involved in breast cancer, we performed an lncRNA microarray analysis in a set of pre-treated tumor tissues from a cohort of breast cancer patient who presented with good or poor responses to chemotherapy. Using a >1.5-fold change and a *P* value of <0.05 as a cutoff point, we found that there were 238 lncRNAs with significantly different expression profile (Supplementary Table 1). The top 10 upregulated and downregulated lncRNAs are shown in Supplementary Figure S1a. In order to investigate the relevance of these lncRNAs in breast cancer development, we first sought to determine their expression levels from TCGA database. As shown in Supplementary Figure S1b, lncRNA ENST00000521030 expression level was upregulated in breast cancer tissues. In order to specify the function of lncRNA-PRLB, we evaluated the expression levels of lncRNA-PRLB and other four unregulated lncRNAs in 18 breast cancer tissues compared with their normal counterparts and breast cancer cells compared with normal breast cells, and found that the differential expression of lncRNA ENST00000521030 was most significant (Fig. 1a, b and Supplementary Figure S1c, d). Therefore, we chose this uncharacterized lncRNA to be a potential candidate to further investigate its role in the progression of breast cancer and named it lncRNA-PRLB. LncRNA-PRLB is located on 8p11.21 in humans and is composed of three exons and has a full length of 663 nt (Supplementary Figure S1e). We then analyzed the coding potential of lncRNA-PRLB. The open reading frame finder of the National Center for Biotechnology Information and conserved domain database failed to predict a protein of more than 55 amino acids based on the lncRNA-PRLB sequence (Supplementary Figure S1f)^25^. In addition, lncRNA-PRLB does not contain a valid Kozak consensus sequence, which further supported the notion that the lncRNA-PRLB has no protein-coding potential. The secondary structure of lncRNA-PRLB is shown in Supplementary Figure S1g. We then analyzed the association between lncRNA-PRLB expression and the clinicopathological features, and they are summarized in Supplementary Table 2. Higher level of lncRNA-PRLB was identified in the primary tumor tissue from patients who developed metastasis (Fig. 1c). Furthermore, Kaplan–Meier survival analysis showed that patients with high lncRNA-PRLB expression levels exhibited poor overall survival and disease-free survival (Fig. 1d and Supplementary Figure S1h). Moreover, high lncRNA-PRLB expressions in tumors and lymph node metastasis had significantly higher risk of death (Supplementary Table 3). Multivariate analysis showed major effects of lncRNA-PRLB overexpression on the patients’ prognosis (Supplementary Table 4). These findings further demonstrated that lncRNA-PRLB may play an important role in the regulation of breast cancer development, and it may serve as a prognostic marker in breast cancer.

### LncRNA-PRLB promotes breast cancer cell proliferation and metastasis in vitro

**Fig. 2:**
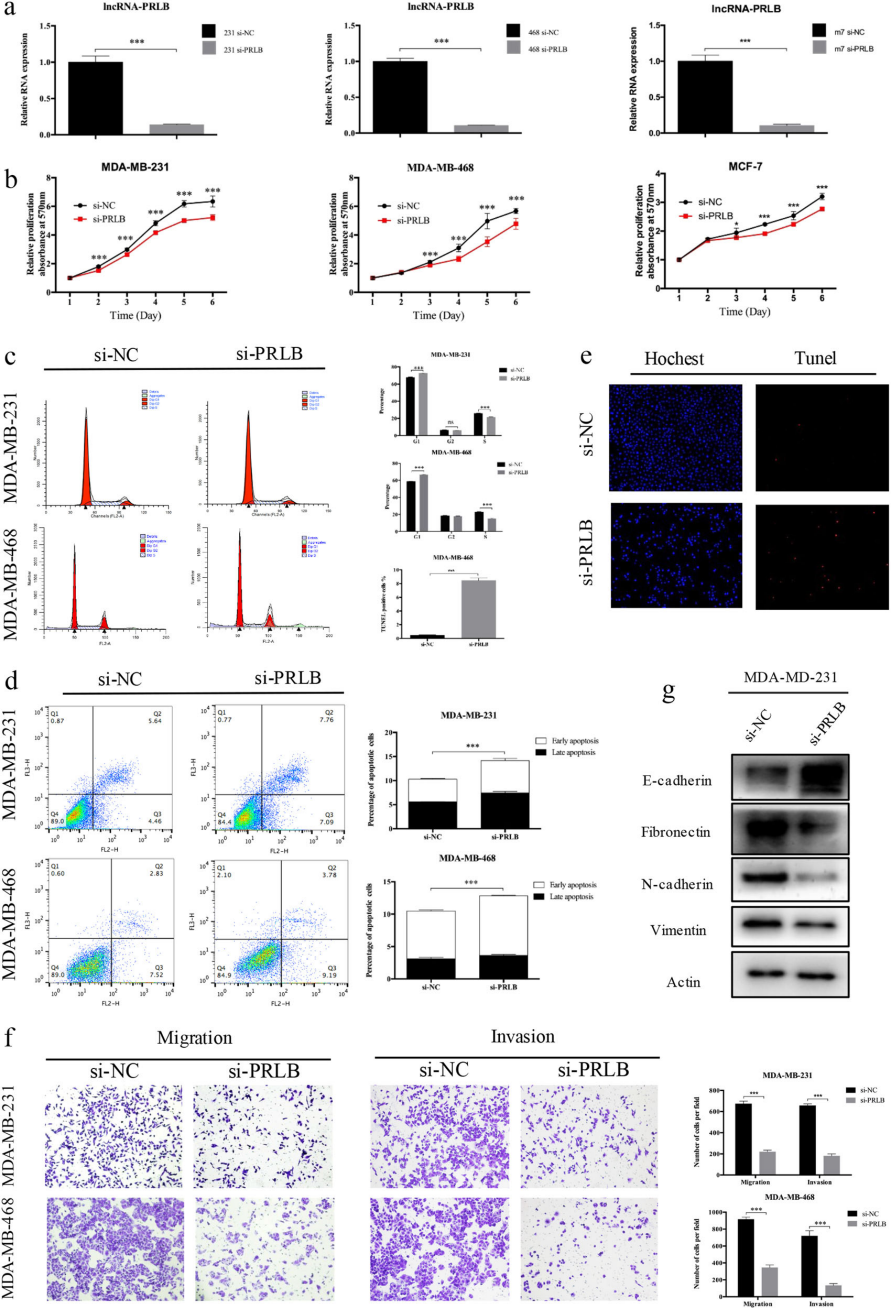
LncRNA-PRLB knockdown inhibited cell proliferation and metastasis in vitro. **a** The knockdown of lncRNA-PRLB in MDA-MB-231, MDA-MB-468, and MCF7 cells was validated with qRT-PCR. **b** Effects of lncRNA-PRLB knockdown on the proliferation of MDA-MB-231, MDA-MB-468, and MCF7 cells were examined with MTT assay. Experiments were performed in triplicate. **c** Flow cytometry was performed to determine the effect of lncRNA-PRLB on changes of cell cycle distribution. The data represent the mean ± SD from three independent experiments. **d** The percentage of apoptotic cells was determined by flow cytometric analysis. The data represent the mean ± SD from three independent experiments. **e** Representative TUNEL staining (red fluorescence) of MDA-MB-468 cells transfected with si-PRLB. Columns are the average of three independent experiments. **f** Transwell migration and invasion assays were used to evaluate MDA-MB-231 and MDA-MB-468 cell numbers with or without lncRNA-PRLB downregulation. Columns are the average of three independent experiments. **g** EMT-related markers were detected by Western blot in MDA-MB-231 cells lines with and without lncRNA-PRLB downregulation. *P < 0.05, **P < 0.01, and ***P < 0.001, Student’s *t* test; NS nonsignificant

We next sought to explore the biological and functional significance of lncRNA-PRLB. The real-time quantitative reverse transcription-PCR (qRT-PCR) analysis was used to detect the transfection efficiency (Fig. 2a). 3-(4,5-Dimethylthiazol-2-yl)-2,5-diphenyltetrazolium bromide (MTT) assays revealed suppressed proliferation in lncRNA-PRLB-downregulated cells (Fig. 2b). Flow cytometry showed that lncRNA-PRLB knockdown increased the G1-phase population, and decreased the S-phase population in three breast cancer cells (Fig. 2c). Compared with negative controls, lncRNA-PRLB downregulation resulted in increased apoptosis in breast cancer cells (Fig. 2d). It was also comparable with the results of terminal-deoxynucleotidyl transferase-mediated nick end labeling (TUNEL) assays (Fig. 2e). Meanwhile, cell migration/invasion assay revealed an inhibited effect on metastasis in cells transfected with si-PRLB (Fig. 2f). Considering the significant role of epithelial–mesenchymal transition (EMT) in cancer cell malignant transformation, we further evaluated the effect of lncRNA-PRLB in EMT. Western blot (WB) showed that lncRNA-PRLB knockdown increased the levels of epithelial markers (E-cadherin) and decreased the levels of mesenchymal markers (N-cadherin, vimentin, and fibronectin) (Fig. 2g). LncRNA-PRLB overexpression could also significantly enhance the proliferation and invasive capacity of breast cancer cells (Supplementary Figure 2). Our findings support the view that lncRNA-PRLB knockdown could inhibit proliferation and invasion in breast cancer cells.

### LncRNA-PRLB is required for 5-FU tolerance of breast cancer cells

**Fig. 3:**
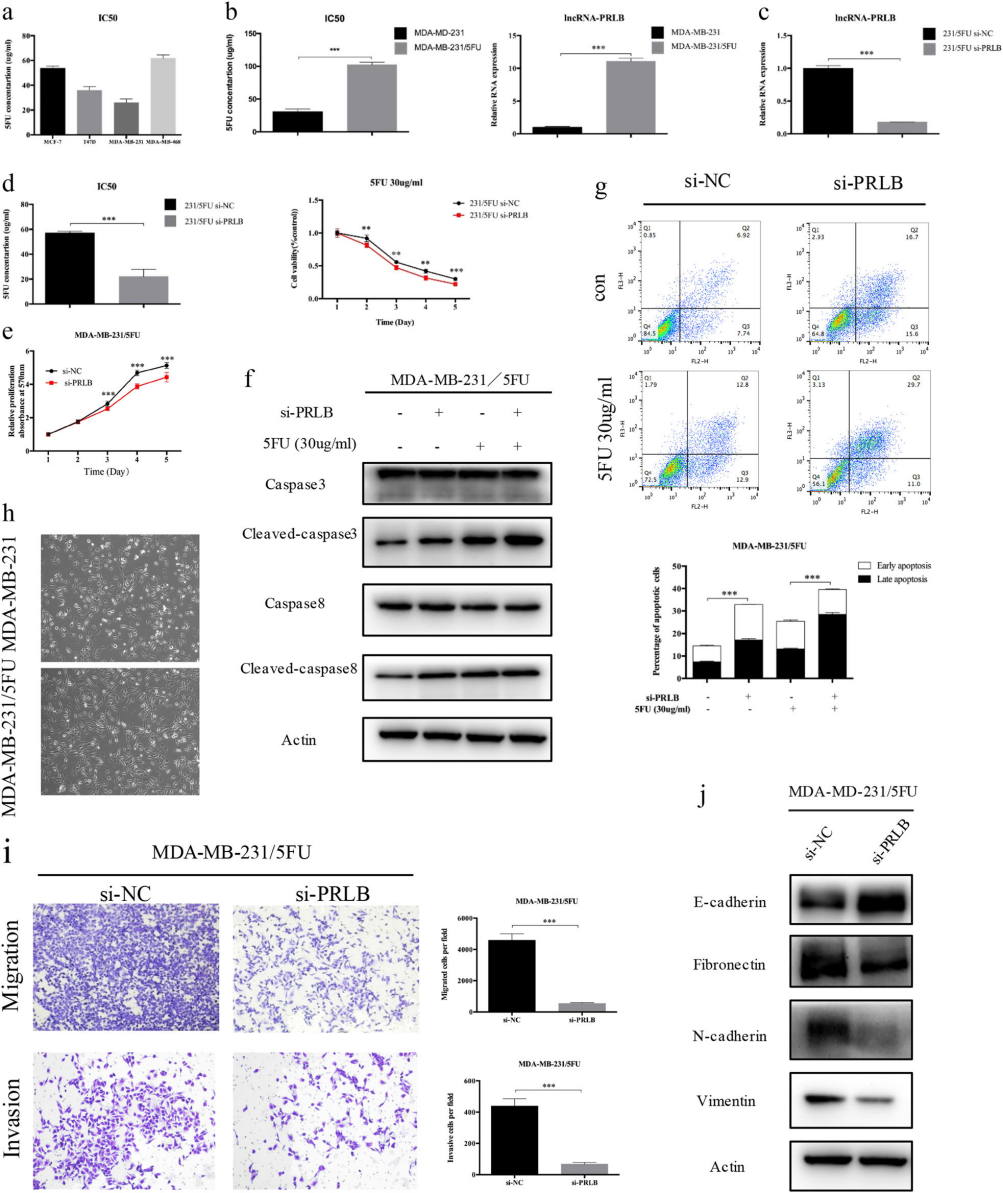
Knockdown lncRNA-PRLB inhibited the chemosensitivity of breast cancer cells to anticancer drugs. **a** The sensitivities of cells to 5-FU were measured by MTT among different breast cancer cells. **b** The IC_50_ values (left) and lncRNA-PRLB expression (right) of MDA-MB-231/5-FU cells was higher than that of their parental cells. **c** Inhibition of lncRNA-PRLB by transfection of si-PRLB in MDA-MB-231/5-FU cells. **d** MTT assays indicated that lncRNA-PRLB knockdown in MDA-MB-231/5-FU cells decreased resistance to 5-FU (left). 5-FU of 30 μg/ml was further used to test the inhibition of drug resistance caused by lncRNA-PRLB knockdown in MDA-MB-231/5-FU cell lines (right). **e** MTT cell proliferation assays performed in MDA-MB-231/5-FU cells transfected with si-PRLB or si-NC. **f** Western blot of apoptosis-related proteins (caspase-3, cleaved-caspase-3, caspase-8, and cleaved-caspase-8). **g** Flow cytometry of MDA-MB-231/5-FU cells transfected with si-PRLB or si-NC, followed by 5-FU treatment. Representative results are shown, and data are presented as mean ± SD. **h** The phenotype of 5-FU-resistant cells and the corresponding parental cells. **i** Transwell assay and **j** Western blot of epithelial markers (E-cadherin) and mesenchymal markers (fibronectin, N-cadherin, and vimentin) were used to measure metastasis capacity in MDA-MB-231/5-FU cells transfected with si-PRLB and si-NC. Columns are the average of three independent experiments. *P < 0.05, **P < 0.01, and ***P < 0.001

Considering the relevance between high metastatic ability of breast cancer cells and the arising of chemoresistance, we further investigated the role of lncRNA-PRLB in 5-fluorouracil (5-FU) resistance. We first evaluated the half-maximal inhibitory concentration (IC_50_) of 5-FU in different breast cancer cells, and MDA-MB-231 showed the lowest resistance to 5-FU (Fig. 3a). Consistently, the expression of lncRNA-PRLB was significantly lower in MDA-MB-231 than other breast cancer cells (Fig. 1c). Thus, we further established 5-FU-resistant cell lines (MDA-MB-231/5-FU). The IC_50_ of 5-FU and lncRNA-PRLB expression in MDA-MB-231/5-FU cells was significantly higher compared with the parental cell lines (Fig. 3b). We suppressed lncRNA-PRLB in MDA-MB-231/5-FU cells (Fig. 3c), and silencing lncRNA-PRLB resensitized breast cancer cells to 5-FU treatment (Fig. 3d). Consistently, lncRNA-PRLB knockdown suppressed cell proliferation (Fig. 3e) and enhanced the 5-FU-induced cell apoptosis, as determined by levels of cleaved-caspase-8 and caspase-3 expressions, as well as flow cytometry (Fig. 3f, g). We further evaluated the effect of lncRNA-PRLB in EMT. MDA-MB-231/5-FU cells presented a fibroblast-like morphology, which is typical of the mesenchymal phenotype of cells compared with the corresponding parental cells (Fig. 3h). Transwell assay revealed that lncRNA-PRLB knockdown inhibited the cell migration ability (Fig. 3i), increased expression of epithelial markers (E-cadherin), and decreased the expression of mesenchymal markers (N-cadherin, vimentin, and fibronectin) (Fig. 3j). Consistently, lncRNA-PRLB overexpression endowed breast cancer cells with refractoriness to 5-FU (Supplementary Figure 3). Consequently, these results indicated that lncRNA-PRLB is required for 5-FU tolerance of breast cancer cells, while enhancing their proliferation and metastasis.

### LncRNA-PRLB abundantly sponges miR-4766-5p and miR-4766-5p promotes cell proliferation, metastasis, and chemoresistance in breast cancer

**Fig. 4:**
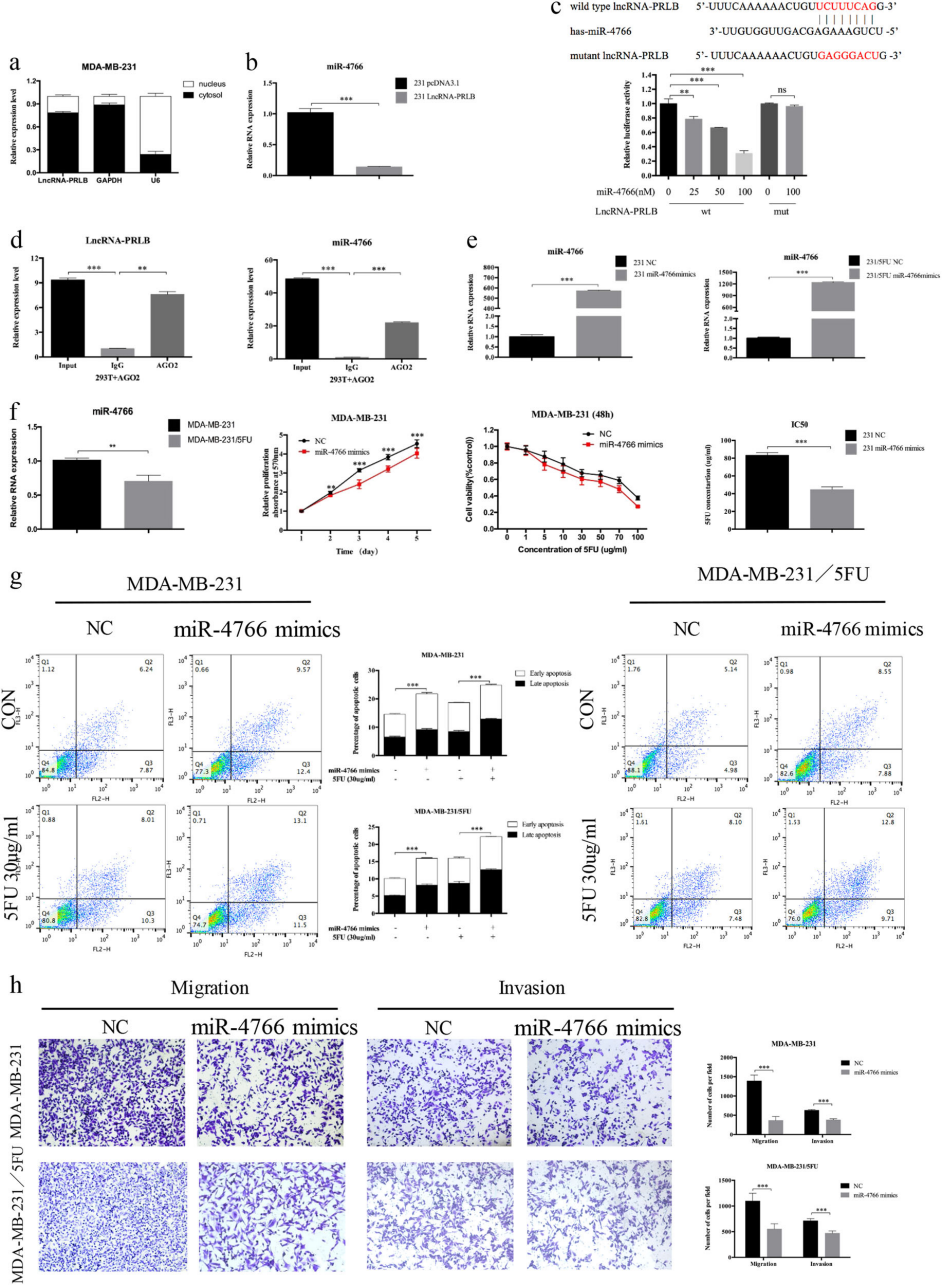
LncRNA-PRLB binds to miR-4766-5p and miR-4766-5p overexpression inhibited cell proliferation, metastasis, and chemoresistance in vitro. **a** qRT-PCR analysis of lncRNA-PRLB nuclear and cytoplasmic expression levels in MDA-MB-231 cells. U6 was used as a nucleus marker, and GAPDH was used as a cytosol marker. **b** qRT-PCR was used to validate the changes of miR-4766 after lncRNA-PRLB overexpression in MDA-MB-231 cells. **c** Schematic illustration of the predicted binding sites between lncRNA-PRLB and miR-4766-5p, and mutation of potential miR-4766-5p-binding sequence in lncRNA-PRLB (left). Luciferase assays in HEK293T cells transfected lncRNA-PRLB wild-type or mutants with miR-4766-5p or NC (right). **d** RIP experiments were performed in HEK293T cells, and the co-precipitated microRNA was subjected to qRT-PCR for lncRNA-PRLB. **e** The overexpression of miR-4766-5p in MDA-MB-231 and MDA-MB-231/5-FU cells was validated with qRT-PCR. **f** Expression levels of miR-4766-5p was determined by qRT-PCR in MDA-MB-231/5-FU cells and their parental cells. MTT cell proliferation assays performed in MDA-MB-231 cells transfected with NC or miR-4766-5p and treated with the indicated concentrations of 5-FU. **g** MDA-MB-231 and MDA-MB-231/5-FU cells were transiently transfected with miR-4766-5p mimics or NC, followed by 5-FU treatment. The apoptosis rates were determined by FACS analysis. Representative results are shown, and data are presented as mean ± SD. **h** Transwell migration and invasion assay were used to measure metastasis capacity in MDA-MB-231 and MDA-MB-231/5-FU cells transfected with miR-4766-5p mimics or NC. *P < 0.05, **P < 0.01, and ***P < 0.001; NS nonsignificant

Many lncRNAs are known to function as a ceRNA to modulate the expression and biological functions of microRNA (miRNA). The subcellular distribution assay revealed that lncRNA-PRLB is predominately located in the plasma (Fig. 4a). The potential targets of lncRNA-PRLB were predicted by the bioinformatics databases (RegRNA); we identified that miR-4766-5p-binding sites were presented in lncRNA-PRLB and the expression level of miR-4766-5p correlated negatively with that of lncRNA-PRLB (Fig. 4b and Supplementary Figure S4a). To further explore the underlying mechanism of the lncRNA/miRNA regulatory function, dual-luciferase reporter assay was performed. MiR-4766-5p mimics reduced the luciferase activity of wild-type lncRNA-PRLB reporter vector, but not that of mutant reporter vector (Fig. 4c). RNA-binding protein immunoprecipitation (RIP) assay was performed to determine whether lncRNA-PRLB and miR-4766-5p are in the same RNA-induced silencing complex. The level of lncRNA-PRLB and miR-4766-5p was higher in anti-Ago2 group than that in the anti-normal IgG group (Fig. 4d). Importantly, miR-4766-5p overexpression caused opposite changes in the expression levels of lncRNA-PRLB (Supplementary Figure S4b), indicating that there exists mutual regulation between miR-4766-5p and lncRNA-PRLB. Taken together, the results indicated that miR-4766-5p was an inhibitory target of lncRNA-PRLB.

**Fig. 5:**
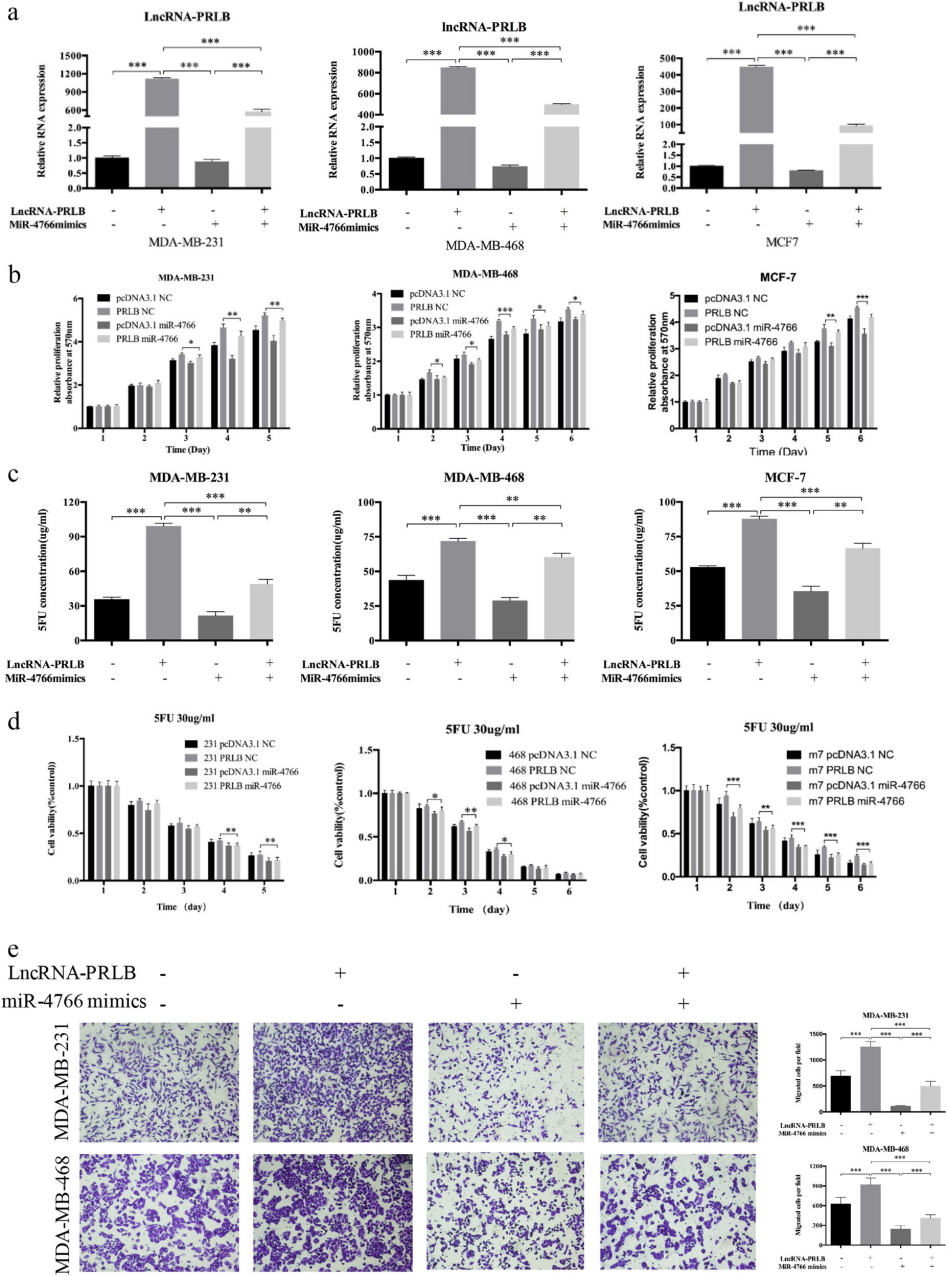
The role of lncRNA-PRLB as a tumor promoter in breast cancer was partially reversed by miR-4766-5p. **a** LncRNA-PRLB expression was analyzed in MDA-MB-231, MDA-MB-468, or MCF7 cells transfected with pcDNA3.1-PRLB or control simultaneously with mimics of miR-4766-5p or NC. **b** MTT assays were used to determine the cell proliferation for pcDNA3.1-PRLB and miR-4766-5p mimics co-transfected MDA-MB-231, MDA-MB-468 and MCF7 cells. **c** MTT assays were used to determine the sensitivity to 5-FU in MDA-MB-231, MDA-MB-468, and MCF7 cells co-transfected with pcDNA3.1-PRLB and miR-4766-5p mimics. **d** 5-FU of 50 μg/ml was used to test the inhibition of drug resistance caused by pcDNA3.1-PRLB and miR-4766-5p mimics co-transfection in MDA-MB-231, MDA-MB-468, and MCF7 cells. **e** Transwell migration assays were used to determine the cell metastasis for pcDNA3.1-PRLB and miR-4766-5p mimics co-transfected MDA-MB-231 and MDA-MB-468 cells. *P < 0.05, **P < 0.01, and ***P < 0.001

To determine the biological function of miR-4766-5p, qRT-PCR was first applied to detect miR-4766-5p expression efficiency (Fig. 4e and Supplementary Figure S4c). Decreased expression of miR-4766-5p was observed in MDA-MB-231/5-FU cells compared with parental cells (Fig. 4f), and miR-4766-5p mimics could inhibit viability, promote apoptosis, inhibit migration, and decrease 5-FU resistance in breast cancer cells (Fig. 4f–h and Supplementary Figure S4d–h). Rescue experiments showed that miR-4766-5p mimics could attenuate the proliferation, migration, and chemoresistance promotion of breast cancer cells induced by lncRNA-PRLB (Fig. 5a–e). The above results suggested that mir-4766-5p negatively regulated proliferation, EMT, and 5-FU resistanceand of breast cancer cells, thereby impaired the function of lncRNA-PRLB.

### miR-4766-5p targets SIRT1 and regulates its expression

**Fig. 6:**
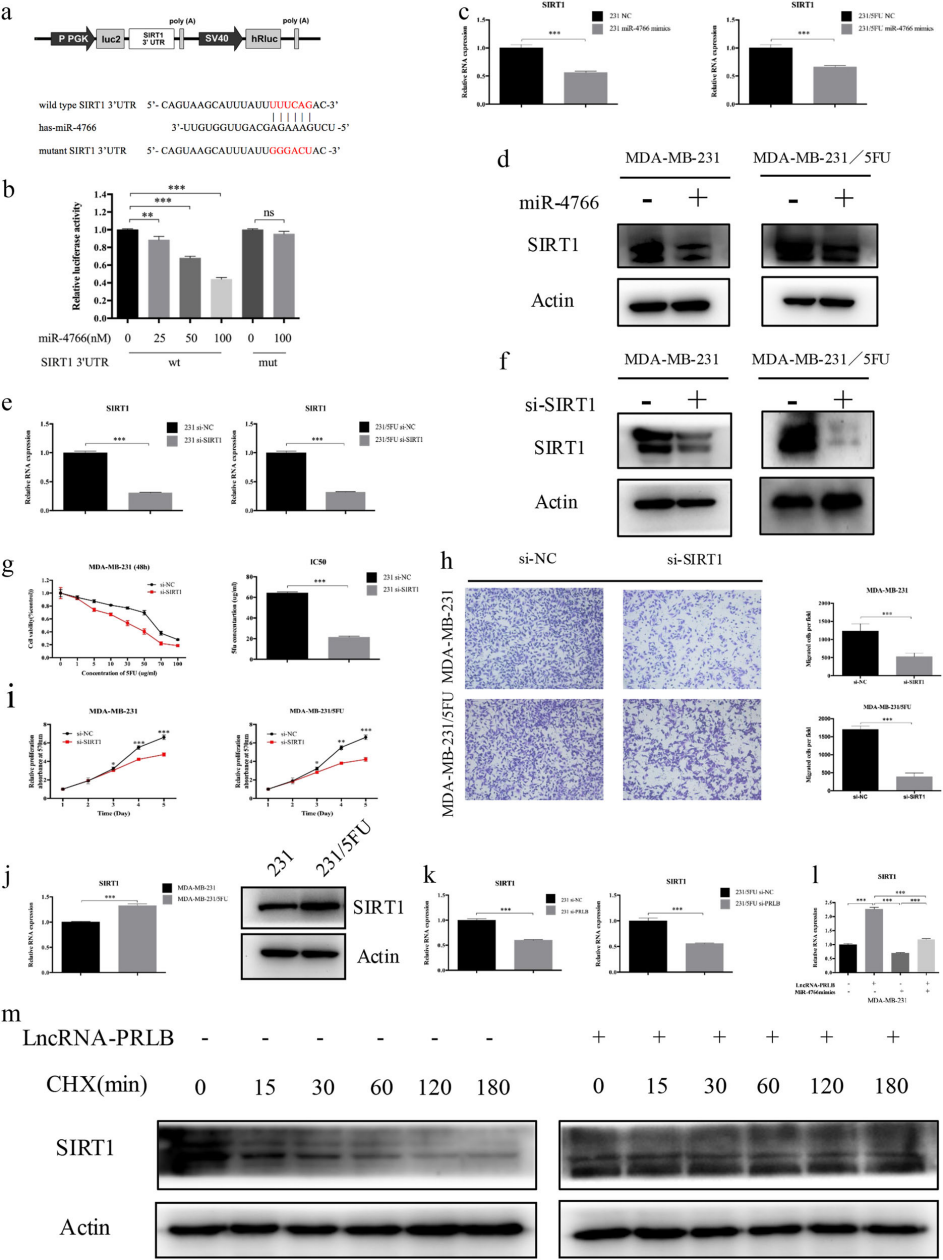
SIRT1 was a target of miR-4766-5p and lncRNA-PRLB, and played a similar role with lncRNA-PRLB. **a** The diagram illustrated the construction of the luciferase reporter plasmids (upper). The schematic graph shows the predicted and mutant binding sites of miR-4766-5p in SIRT1 3′-UTR (lower). **b** The relative luciferase activity in HEK293T cells with and without miR-4766-5p overexpression was measured when transfected with WT or MUT luciferase plasmids. **c** qRT-PCR and **d** Western blot analysis revealed miR-4766-5p overexpression significantly reduced SIRT1 expression in different cell lines. **e** qRT-PCR and **f** WB were used to validate the knockdown of SIRT1 in MDA-MB-231 and MDA-MB-231/5-FU. **g** MTT cell proliferation assay performed in MDA-MB-231 cells transfected with si-SIRT1 or si-NC and treated with the indicated concentrations of 5-FU. Columns are the average of three independent experiments. **h** Transwell migration assay was used to measure metastasis capacity in MDA-MB-231 and MDA-MB-231/5-FU cells transfected with si-SIRT1 or si-NC. **i** MTT cell proliferation assays performed in MDA-MB-231 and MDA-MB-231/5-FU cells transfected with si-SIRT1 or si-NC. **j** qRT-PCR and WB were used to determine the expression level of SIRT1 in MDA-MB-231/5-FU cells and their parental cells. **k** LncRNA-PRLB knockdown downregulated SIRT1 levels in MDA-MB-231 and MDA-MB-231/5-FU cells. **l** qRT-PCR was used to determine the SIRT1 expression level for pcDNA3.1-PRLB and miR-4766-5p mimics co-transfected MDA-MB-231 cells. **m** MDA-MB-231 cells were transfected with pcDNA3.1-PRLB or control constructs. At 48 h after transfection, cells were treated with 50 μg/ml cycloheximide (CHX) for the indicated lengths of time. Equal amounts of cell lysates were detected by Western blotting. *P < 0.05, **P < 0.01, and ***P < 0.001

Using the bioinformatics algorithms TargetScan, we identified that miR-4766-5p potentially targeted SIRT1. Dual-luciferase reporter assays found that miR-4766-5p mimics led to the attenuation of fluorescence of the wild-type 3′-untranslated region (WT 3′-UTR), but had no effect on the mutant (MUT) 3′-UTR of SIRT1 (Fig. 6a, b). Furthermore, the protein levels of SIRT1 protein and RNA in breast cancer cells were reduced by miR-4766-5p overexpression (Fig. 6c, d and Supplementary Figure S5a, b). These results indicate that SIRT1 is a direct target of miR-4766-5p.

### LncRNA-PRLB promotes proliferation, metastasis, and chemoresistance partly by regulating SIRT1 expression through protecting it from miR-4766-5p-mediated degradation

To clarify the role of SIRT1 in breast cancer cells, we perturbed SIRT1 levels in breast cancer cells. The efficiency of SIRT1 knockdowns were confirmed by qRT-PCR and WB (Fig. 6e, f). After SIRT1 knockdown, we observed significant decrease in cell proliferation, motility, and 5-FU resistance (Fig. 6g–i). Moreover, increased expression of SIRT1 was observed in 5-FU-resistant cell lines compared with the parental cell lines (Fig. 6j). We also determined whether lncRNA-PRLB regulated SIRT1 expression in breast cancer cells. WB and qRT-PCR showed that SIRT1 expression was downregulated in cells transfected with si-PRLB and was upregulated in lncRNA-PRLB-overexpressed cells (Fig. 6k and Supplementary Figure S6a, b). Interestingly, 5-FU treatment could further upregulate SIRT1 protein level in lncRNA-PRLB-overexpressed cells, but showed no important effect in cells transfected with si-PRLB (Supplementary Figure S6c, d), indicating that the increased expression of SIRT1 caused by 5-FU treatment was partly dependent on lncRNA-PRLB. Significantly, while lncRNA-PRLB overexpression led to increased expression of SIRT1, simultaneous miR-4766-5p overexpression was able to reverse the promotion of SIRT1 expression (Fig. 6l and Supplementary Figure S6e). Moreover, cycloheximide, an inhibitor of protein biosynthesis, could decrease the level of SIRT1 over time. Remarkably, lncRNA-PRLB overexpression could significantly increase the half-life of SIRT1 (Fig. 6m). Collectively, these data strongly support the hypothesis that lncRNA-PRLB promotes breast cancer cell proliferation and chemoresistance via miR-4766-5p-mediated regulation of SIRT1 signaling.

### LncRNA-PRLB promotes breast cancer cell proliferation, metastasis, and chemoresistance in vivo

**Fig. 7:**
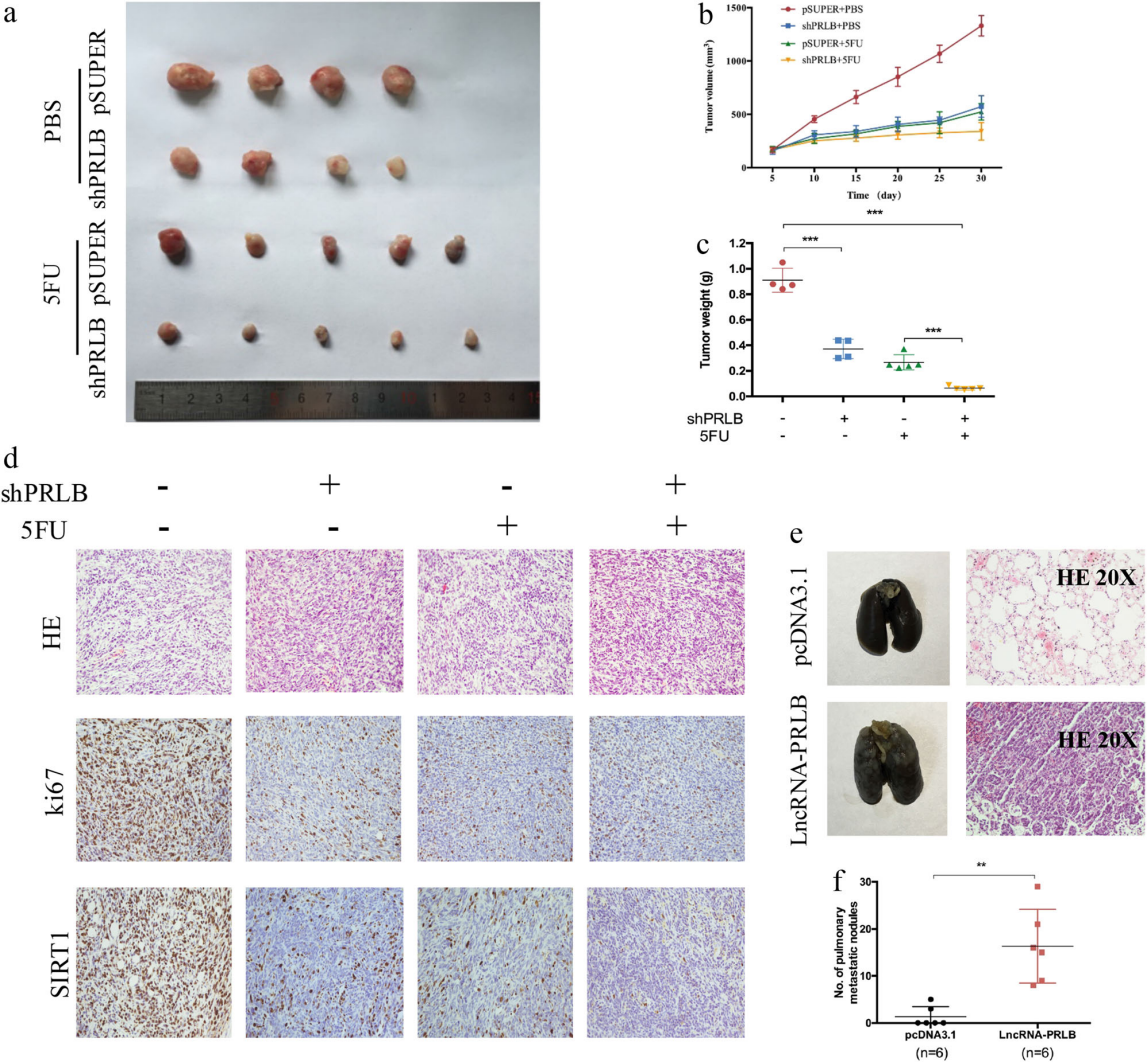
LncRNA-PRLB promoted tumorigenesis, metastasis, and chemoresistance in vivo. MDA-MB-231 cells were transfected with shPRLB or pSUPER and injected subcutaneously into nude mice. When the average tumor size reached approximately 100 mm^3^, 5-FU was administered through intraperitoneal injection at a dose of 50 mg/kg every other day for three doses in total. **a** Photographs illustrated tumors in xenografts. **b** Growth curve of xenograft tumors after the first administration of 5-FU. **c** LncRNA-PRLB knockdown combined with 5-FU treatment resulted in a dramatic decline of tumor weight. **d** Representative images of H&E and immunohistochemical staining of the tumor. IHC revealed a downregulation of the proliferation index Ki-67 and SIRT1. **e** Representative images of lungs and hematoxylin and eosin (H&E) staining of lungs isolated from mice that received tail vein injection of MDA-MB-231 pcDNA3.1-PRLB cells and MDA-MB-231 pcDNA3.1 cells. Each group contains six mice. **f** The number of pulmonary metastatic nodules in the lung were counted and analyzed with Student’s *t* test. *P < 0.05, **P < 0.01, and ***P < 0.001

We then used a nude mouse xenograft model to further investigate the oncogenic role of lncRNA-PRLB in vivo. MDA-MB-231 cells stably transfected with shPRLB or control were subcutaneously injected into mice. The shPRLB group treated with phosphate-buffered saline (PBS) or 5-FU showed significantly reduced tumor growth and tumor weight compared with the controls (Fig. 7a–c). Moreover, combined treatment with lncRNA-PRLB knockdown and 5-FU led to an even further reduction in tumor volume and tumor weight (Fig. 7a–c). Consistently, lncRNA-PRLB and SIRT1 expression were lower, while miR-4766-5p expression was higher in tumor tissues derived from the shPRLB group than those from controls (Supplementary Figure S7a). Moreover, the hematoxylin and eosin (H&E) staining and immunohistochemistry assay results showed that the tumors developed from shPRLB cells displayed alterations in shape and reduced Ki-67 and SIRT1 staining compared with tumors formed from empty vector-transfected cells (Fig. 7d).

To further evaluate the effect of lncRNA-PRLB on tumor metastasis in vivo, MDA-MB-231 cells with stable lncRNA-PRLB overexpression and control cells were injected into nude mice through the tail vein. H&E staining was performed to evaluate tissue morphology (Fig. 7e). All six mice transplanted with MDA-MB-231 lncRNA-PRLB cells had small pulmonary metastatic nodules, while only two out of six mice in the control group ended up with lung metastasis. Moreover, both the volume and number of lung metastatic nodules were increased in lncRNA-PRLB overexpression groups compared with control groups (Fig. 7e, f). Our findings indicate that lncRNA-PRLB had an important role in breast cancer tumor development and chemoresistance in vivo.

## Discussion

Accumulating studies have reported that lncRNAs are dysregulated in many types of human cancers, including breast cancer^26^, bladder cancer^27^, prostate cancer^28^, colorectal cancer^20^, ovarian cancer^29^, osteosarcoma^30^, and so on, providing novel therapeutic opportunities to treat cancer. Considering the abundance and functionality of lncRNAs, it is worthwhile to systematically explore the function of those uncharacterized RNA transcripts, which may help exploit new promising therapeutic strategy for the treatment of breast cancer.

Our studies revealed that lncRNA-PRLB, a novel lncRNA located in chromosome 8, played essential role in breast cancer progression and chemoresistance. LncRNA-PRLB was significantly upregulated in breast cancer cell lines and breast cancer tissues. High lncRNA-PRLB expression was associated with worse prognosis, and lncRNA-PRLB expression was an independent prognostic factor for breast cancer patients. However, we failed to evaluate the influence of metastasis on overall survival in multivariate analysis, possibly due to the shorter follow-up time and smaller size of the cohort. Then, we further explore the functional role of lncRNA-PRLB in breast cancer. Our data indicated that knockdown of lncRNA-PRLB inhibited and overexpression of it promoted breast cancer cell proliferation, metastasis, chemoresistance, and tumor growth both in vitro and vivo, suggesting that lncRNA-PRLB functions as an oncogene. Accumulating evidence suggested that tumor metastasis and chemoresistance might be closely associated to biological events, which are two major inseparable causes of cancer-related lethality^31, 32^. Our results demonstrated that lncRNA-PRLB knockdown could significantly inhibit the migration and invasion ability in both MDA-MB-231/5-FU and parental cells, preliminarily revealing the relevance between metastasis and chemoresistance. In response to chemotherapy, cell apoptosis was one of the most commonly activated pathway, and disruption of apoptosis would facilitate multidrug resistance. 5-FU is a pyrimidine analog, which works through irreversible inhibition of thymidylate synthase and results in apoptosis of cancer cells^33^. In this manuscript, we showed that lncRNA-PRLB knockdown could significantly increase apoptosis after 5-FU treatment, and lncRNA-PRLB alone also showed a slight effect on the apoptosis pathway, indicating that lncRNA-PRLB knockdown could enhance the susceptibility of breast cancer cells to 5-FU via increasing 5-FU-induced apoptosis. Although there was a significant effect of lncRNA-PRLB on 5-FU resistance, investigation on more types of chemotherapy was needed to enhance the significance of lncRNA-PRLB on chemoresistance. Recent studies showed that tumor microenvironment (TME) played significant role in proliferation, metastasis, and chemoresistance of cancer cells^34^. And, modulation or disruption in TME could lead to a more effective management of cancer. Many lncRNAs were found to be involved in TME. Linc00092 is upregulated in response to the chemokine in ovarian cancer cells^35^. LncRNA-MALAT1 promoted FGF2 protein secretion in tumor-associated macrophage, leading to inhibited inflammatory cytokine release, and thus promoting the proliferation and metastasis of thyroid cancer^36^. Therefore, other functions of lncRNA-PRLB, such as regulation in immune effector cells, release of cancer immunoediting, macrophage polarization, and so on, would be investigated in future work. Thus, our present study identified lncRNA-PRLB as a potential prognostic marker and a therapeutic target for breast cancer.

To further investigate the mechanisms of lncRNA-PRLB, we first explored its location in breast cancer cells. Cell cytoplasm/nucleus fraction isolation assay identified that lncRNA-PRLB was mainly located in the cytoplasm. Then, ceRNA mechanism was first considered^37^. Multiple studies have documented that lncRNAs harbor potential miRNA-responsive elements and function as competitive platforms for miRNAs in multiple types of cancer, thus reducing the repression of mRNAs^38–40^. Through bioinformatic algorithms, we predicted a novel microRNA, miR-4766-5p, as a potential target of lncRNA-PRLB. Dual-luciferase reporter gene assay revealed the direct interaction between lncRNA-PRLB and miR-4766-5p, which was also proven by co-immunoprecipitation with the Ago2 protein in human embryonic kidney 293T (HEK293T) cells^38, 41^. We also found that changes in miR-4766-5p expression led to opposite changes in the expression of lncRNA-PRLB, and miR-4766-5p could partly abolish the lncRNA-PRLB-mediated biological effect. Moreover, lncRNA-PRLB regulated the expression of the miR-4766-5p target gene, *SIRT1*. On the other hand, overexpression of miR-4766-5p reduced the lncRNA-PRLB expression, suggesting that lncRNA-PRLB and miR-4766-5p could form a reciprocal repression feedback loop^42^. The direct binding of miR-4766-5p and 3′-UTR of SIRT1 was further validated by dual-luciferase reporter assay. In addition, our in vitro and in vivo systems revealed that the lncRNA-PRLB knockdown led to a decrease in the expression of SIRT1 and an increase in the expression of miR-4766-5p. Together, these data strongly supported the role of lncRNA-PRLB as a sponge, or ceRNA, for miR-4766-5p, and we firstly identified miR-4766-5p as a tumor suppressor in breast cancer.

SIRTs are a family of NAD^+^-dependent class III histone deacetylases^43, 44^, including seven human SIRTs with diverse subcellular locations and functions. SIRT1 is the most extensively studied member among SIRT family. The expression of SIRT1 is upregulated in multiple types of tumors, and is associated with tumor progression and resistance to cancer chemotherapy^45, 46^. Zhang et al.^47^ revealed that SIRT1 overexpression was associated with poor survival outcome in gastric cancer patients. Silencing SIRT1 suppressed non-small-cell lung cancer cell proliferation and dramatically suppressed tumor formation^48^. SIRT1 was significantly upregulated in pancreatic cancer tissues and cell lines, and played a role in the regulation of pancreatic cancer cell proliferation and migration^49^. Another study also showed that SIRT1 expression levels were significantly upregulated in breast cancer tissues, and SIRT1 overexpression eliminated the suppressive effects of the miR-22 overexpression on the malignant phenotype of MCF7 cells^50^. Interestingly, some studies claimed that SIRT1 could inhibit tumor progression. SIRT1 overexpression inhibited human colon cancer cell proliferation^51^. Ectopic expression of SIRT1 in mesenchymal stem cells suppressed breast cancer growth^52^, while reduced SIRT1 levels in breast cancer cells led to increased metastasis through deacetylating Smad4 signaling^53^. According to previous studies, it remains controversial whether SIRT1 acts as an oncogene or tumor suppressor, and its specific functions may depend on diverse biological signaling. In addition, researches on SIRT1 in breast cancer were particularly confusing. Here, we demonstrated that SIRT1 knockdown could inhibit proliferation, metastasis, and chemoresistance in breast cancer cells, which was modulated by lncRNA-PRLB/miR-4766-5p network. Therefore, our study appears to support a tumor promoter role for SIRT1 in breast cancer. Moreover, lncRNA-PRLB could not only increase SIRT1 at the mRNA level but also at the protein level. We further proved that lncRNA-PRLB overexpression could dramatically prolong the half-life of SIRT1 protein after adding the translation inhibitor cycloheximide, indicating the effect of lncRNA-PRLB on the protein stability of SIRT1.

In conclusion, the present study for the first time revealed that lncRNA-PRLB was upregulated in breast cancer tissues and cell lines, and higher levels of lncRNA-PRLB were associated with tumor progression and inversely correlated with prognosis. It was reasonable for us to conclude that lncRNA-PRLB promoted breast cancer cell proliferation, metastasis, and chemoresistance via miR-4766-5p-mediated regulation of SIRT1 signaling, although more clinical samples and further investigations of the exact roles of lncRNA-PRLB in other cancers were required. On the basis of these findings, the present study provided a better understanding of the lncRNA–miRNA feedback loop function in breast cancer progression. LncRNA-PRLB could be considered as a potential target for the breast cancer therapies in the future.

## Materials and methods

### Patients and samples

Tumor samples were obtained from breast cancer patients admitted to Qilu hospital from January 2004 to December 2011. The period of follow-up of all patients is 1–96 months, with a median of 43 months. For all participants in this study, written informed consent for the use of these clinical materials in research was obtained. This study was approved by the Ethical Committee of Shandong University.

### Microarray analysis

LncRNA microarrays were used to profile lncRNA expression from two chemosensitive breast cancer tissues and two chemoresistant tissues. GeneChips were scanned by the Affymetrix^®^ GeneChip Command Console. Quantile normalization and subsequent data processing were analyzed by Agilent Gene Spring Software 12.6 (Agilent Technologies, Santa Clara, CA, USA). A *P* value was calculated using the paired *t* test. When the lncRNA expression level changed at least 1.5-fold with *P* < 0.05, the lncRNA expression was then considered significantly different.

### Cell lines and culture conditions

All the cell lines were purchased from American Type Culture Collection (ATCC, Manassas, VA, USA) and were routinely maintained in Dulbecco's modified Eagle's medium/high glucose medium (Gibco-BRL, Rockville, IN, USA) with 10% fetal bovine serum, 100 U/mL penicillin, and 100 μg/mL streptomycin in a humidified incubator under the conditions of 5% CO_2_ at 37 °C.

### RNA extraction, reverse transcription, and qRT‐PCR

Total RNA was extracted from cells or tissues using TRIzol reagent (Invitrogen, Carlsbad, CA, USA). For miRNA, RNA was converted into cDNA using PrimeScript miRNA cDNA Synthesis Kit (TaKaRa, Shiga, Japan). PrimeScript reverse transcriptase (RT) Reagent Kit (TaKaRa, Shiga, Japan) was used to synthesize cDNA of lncRNA and mRNA. qRT-PCR was performed using a SYBR green PCR mix in Applied Biosystems 7900HT Real-Time PCR System. The primers (Sangon Biotech, Shanghai, China) used in this study were listed in Supplementary Table 5. β-Actin was used as an endogenous control for mRNA expression of each sample and small nuclear RNA U6 for the expression of microRNAs. Experiments were repeated three times to confirm the findings. Gene expression profiles were calculated using 2^−ΔΔCT^ method.

### Psmid construction and transfection

For overexpression of lncRNA-PRLB, lncRNA-PRLB cDNA was cloned into the multiple cloning site of the pcDNA3.1 vector (Invitrogen, Carlsbad, CA, USA). Both the expression plasmid vector and the empty vector were used to transfect breast cancer cells using Lipofectamine 2000 (Invitrogen, MA, USA) to establish lncRNA-PRLB overexpression and control cell lines, screened with puromycin (2 μg/ml) for 4 weeks. LncRNA-PRLB knockdown cell lines were transfected with pSUPER or shPRLB. Transfection was performed with Lipofectamine 2000 (Invitrogen) according to the manufacturer’s instructions. All the mimics or siRNA were purchased from Applied Biological Materials (ABM, Canada).

### MTT and in vitro chemosensitivity assay

Cells were cultured in 96-well plates in the culture medium. After incubation overnight, the medium was replaced with solutions containing different concentrations of 5-FU with the indicated concentration for 48 h or 30 μg/ml 5-FU followed by incubation for the indicated time. Afterwards, 20 μl of MTT (5 mg/ml in PBS) was added to each well and the cells were incubated for another 4 h at 37 °C. The supernatants were aspirated carefully and then 100 μl of dimethyl sulfoxide was added to each well. The absorbance values were read using a Microplate Reader (Bio-Rad, Hercules, CA, USA) at 570 nm after gently shaking of the plate for 10 min at room temperature.

### Cell cycle assay

After 48 h of transfection, cells were trypsinized, and then washed with cold PBS twice. Then, the cell plates were suspended with 300 μl PBS, followed by the addition of 500 μl cell cycle staining buffer (MultiSciences (Lianke) Biotech Co., Ltd.). After 30 min incubation at room temperature, cells were sorted by FACS Calibur (BD Biosciences, Franklin Lakes, NJ, USA), and the profiles were analyzed by the ModFitLT V2.0 software (Becton Dickinson).

### PE Annexin V apoptosis assay

PF/Annexin V Appoptosis Detection Kit (BD Biosciences, Franklin Lakes, NJ, USA) was used for apoptosis analysis following the manufacturer’s instruction. 5-FU of 30 μg/ml was used to induce apoptosis for 24 to 48 h. Cells were collected, washed twice with PBS, and gently re-suspended in 500 μl binding buffer. After the addition of 5 μl Annexin V-FITC and 5 μl propidium iodide, the cells were incubated in the dark for 15 min at room temperature, followed by immediate flow cytometry analysis on a FACS Calibur (BD Biosciences, Franklin Lakes, NJ, USA).

### TUNEL assay

5-FU of 30 μg/ml was used for treatment. TUNEL Apoptosis Assay was performed using the One Step TUNEL Apoptosis Assay Kit (Beyotime, Jiangsu, China) according to the guidelines. Apoptotic cells (red fluorescence) were imaged under a fluorescent microscopy.

### WB analysis

Total cellular protein from each sample were separated by 10% sodium dodecyl sulfate-polyacrylamide gel electrophoresis and electroblotted onto a polyvinylidene fluoride membrane using a semidry blotting apparatus (Bio-Rad, Hercules, CA, USA). The membranes were blocked with 5% nonfat milk at room temperature for 1 h, followed by incubation with primary antibodies (Immuno-Way, Newark, DE, USA) overnight at 4 °C. After washing with TBST and incubation with the appropriate secondary antibodies, the protein bands were detected using the Pro-lighting HRP agent. Expression of β-actin was used as an endogenous loading control.

### Migration and invasion assays

Migration and invasion assays were performed using the Transwell system (24-wells, 8-μm pore size with polycarbonate membrane; Corning Costar, Lowell, MA, USA). Cells (1 × 10^5^ cells per well) were plated on the top side of polycarbonate Transwell filter coated (for invasion assays) or uncoated (for migration assays) with Matrigel in the top chamber. Cells were suspended in the medium without serum, and the medium supplemented with serum was used as a chemoattractant in the bottom chamber. The cells were incubated at 37 °C for 24 h. The noninvasive cells in the top chambers were removed with cotton swabs, whereas the migrated and invaded cells on the lower membrane surface were fixed in 100% methanol for 10 min, and then stained with 0.2% Giemsa and counted in three random fields using an Olympus light microscope.

### Subcellular fractionation location

The separation of nuclear and cytosolic fractions was performed using the PARIS Kit (Life Technologies, Carlsbad, CA, USA) according to the manufacturer’s instructions.

### Luciferase assay

LncRNA-PRLB with WT or MUT miR-4766-5p-binding sites were generated and fused to the luciferase reporter vector pmirGLO (Promega, Madison, WI, USA). The full-length WT 3′-UTR containing the predicted miR-4766-5p targeting site, and MUT 3′-UTR of SIRT1 were amplified and cloned into the pmirGLO vector. HEK293T cells were placed on a 96-well plate and co-transfected with luciferase plasmids and miR-4766-5p or control miRNA. After 48 h transfection, luciferase activity was measured with the dual-luciferase reporter assay system (Promega). Firefly luciferase activity was normalized against Renilla luciferase activity.

### RIP assay

RIP experiments were performed using the Magna RIP RNA-Binding Protein Immunoprecipitation Kit (Millipore, Billerica, MA, USA) according to the manufacturer’s instructions. Antibody for RIP assays of AGO2 or control IgG was from Millipore. The co-precipitated RNAs were detected by qRT-PCR. The total RNAs were the input controls.

### Tumor xenograft experiments

Stably transfected cells were harvested and re-suspended in serum-free medium at a concentration of 1 × 10^7^ cells/0.2 ml, and injected subcutaneously into each flank of the 4–6-week-old nude female mice. When the average tumor size reached approximately 100 mm^3^, 5-FU was administered through intraperitoneal injection at a dose of 50 mg/kg every other day for three doses in total. Tumor size was monitored every 5 days, and mice were euthanized after 30 days. Both maximum (*L*) and minimum (*W*) length of the tumor were measured using a slide caliper, and the tumor volume was calculated as ½*LW*^2^. To produce experimental lung metastasis, 5 × 10^5^ cells were injected into the lateral tail veins of female nude mice (six mice per group). After 4 weeks, all the mice were killed under anesthesia. The lungs were collected and fixed in 10% formalin and embedded in paraffin. H&E staining was performed on sections from embedded samples.

### Immunohistochemical analysis

Tissue sections were dried at 60 °C for 1 h, and then dewaxed in xylene and rehydrated in a graded series of ethanol. Antigen retrieval was performed by microwave heating. Nonspecific antigens were blocked with 5% bovine serum albumin in the room temperature for 30 min. Each section was treated with Ki-67 rabbit polyclonal antibodies (1:100 solution) at 4 °C overnight. Each section was incubated with secondary antibody at 37 °C for 1 h, then stained with diaminobenzidine, and counterstained with hematoxylin. The representative areas were selected for images using an Olympus light microscope

### Statistical analysis

SPSS version 18.0 software was used for statistical analysis. Data are presented as the mean ± standard error of the mean (SEM) from at least three independent experiments. Two group comparisons were performed with a Student’s *t* test. Survival rates were calculated by Kaplan–Meier survival analysis. Cox proportional hazards model multivariate analyses were used to evaluated the influence of lncRNA-PRLB expression and clinicopathological features on overall survival. Differences with *P* < 0.05 were considered statistically significant.

## Electronic supplementary material

Supplementary Figure and Figure Legend

Supplementary Table I

Supplementary Table II III IV V
