# Supplementary material for: A novel long non-coding RNA-PRLB acts as a tumor promoter through regulating miR-4766-5p/SIRT1 axis in breast cancer
Source: Cell Death Dis. 2018 May 11;9(5):563. doi: 10.1038/s41419-018-0582-1 (PMC5948209; doi:10.1038/s41419-018-0582-1)
Supplement: Supplementary file 1 — Supplementary Figure and Figure Legend [file 41419_2018_582_MOESM1_ESM.docx]

**Supplementary Figure**

**
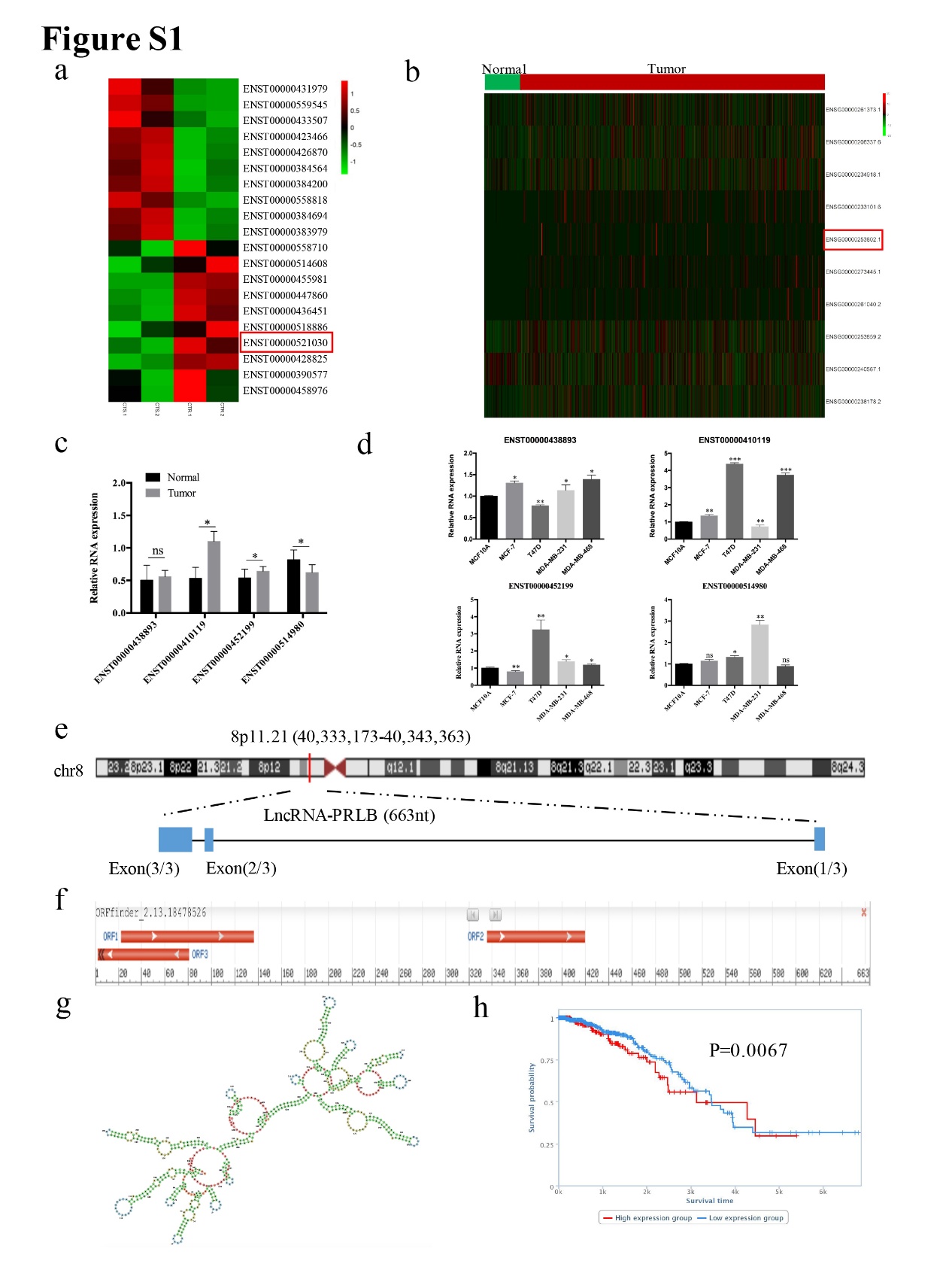
**

**Supplementary Figure S1 LncRNA-PRLB expression level was upregulated in breast cancer cancer and associated with prognosis of breast cancer patients.** (a) LncRNA-PRLB was upregulated in breast cancer patients that presented with poor responses to chemotherapy. (b) LncRNA-PRLB was upregulated in breast cancer patients (TCGA). (c) The relative expression of indicated four lncRNAs in breast cancer tissues (n=18) compared with corresponding non-tumor tissues (n=18). (d) The relative expression of indicated four lncRNAs in breast cancer cell lines and normal breast epithelial cell line MCF-10A. (e) Schematic annotation showed lncRNA-PRLB genomic locus in humans. Blue rectangles represent exons. (f) Putative proteins possibly encoded by lncRNA-PRLB as predicted by the ORF Finder. (g) The secondary structure of lncRNA-PRLB got from NOCODE. (h) High expression of lncRNA-PRLB predicted a shorter overall survival (P = 0.0067) in primary breast cancer samples.


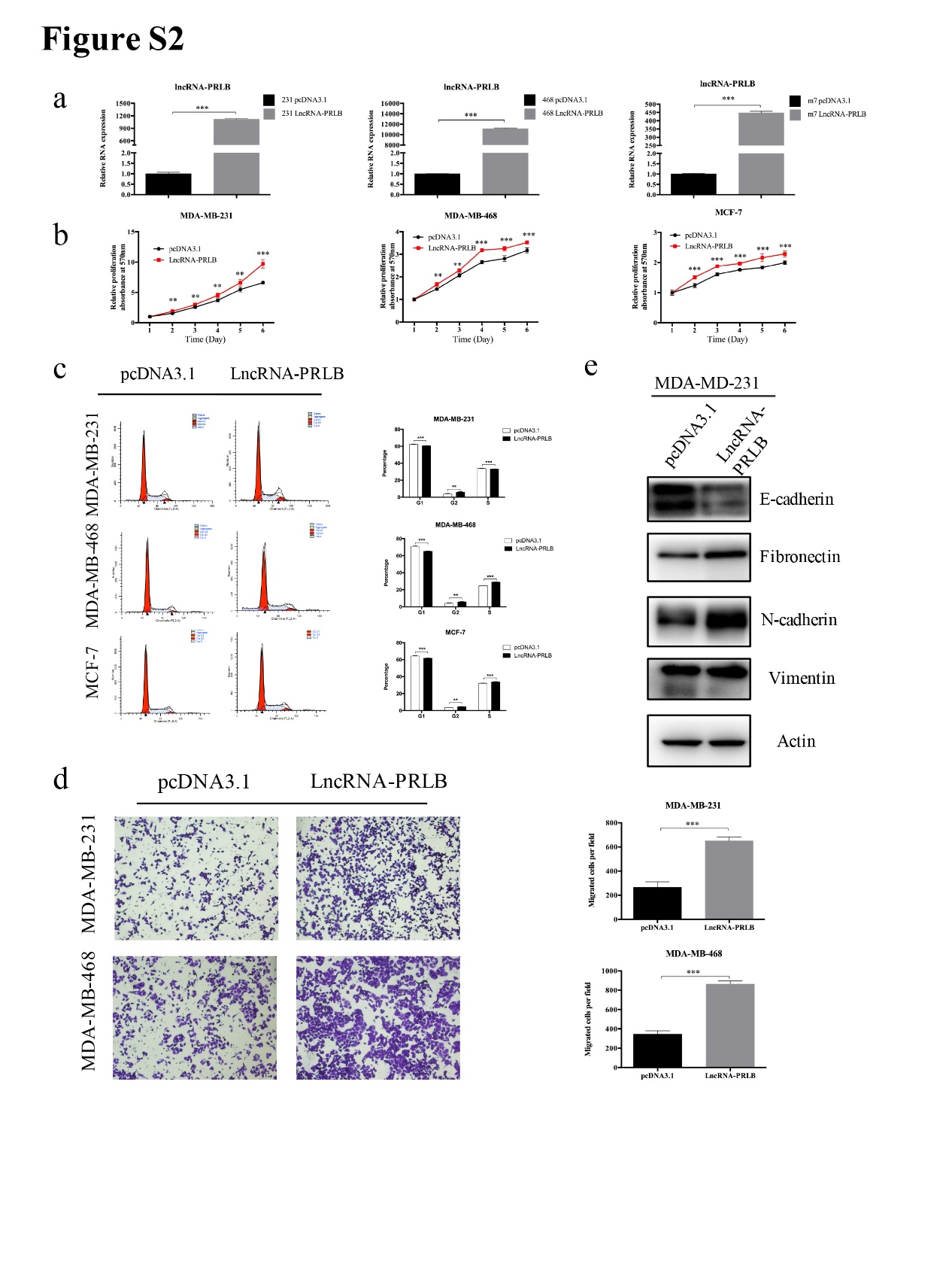


**Supplementary Figure S2 LncRNA-PRLB overexpression promoted proliferation and migration in vitro.** (a) The overexpression of lncRNA-PRLB in MDA-MB-231, MDA-MB-468 and MCF-7 cells was validated with qPCR. (b) Effects of lncRNA-PRLB overexpression on the proliferation of MDA-MB-231, MDA-MB-468 and MCF-7 cells were examined with MTT assay. Experiments were performed in triplicate. (c) Flow cytometry was performed to determine the effect of lncRNA-PRLB on changes of cell cycle distribution. The data represent the mean ± S.D. from three independent experiments. (d) Transwell migration assays demonstrated that lncRNA-PRLB overexpression promoted cell migration abilities. (e) Western blotting showed that lncRNA-PRLB overexpression led to decreased expression of E-cadherin and increased expression of Fibronectin, N-cadherin and vimentin. (*P<0.05, **P<0.01, ***P<0.001, Student’s t test)


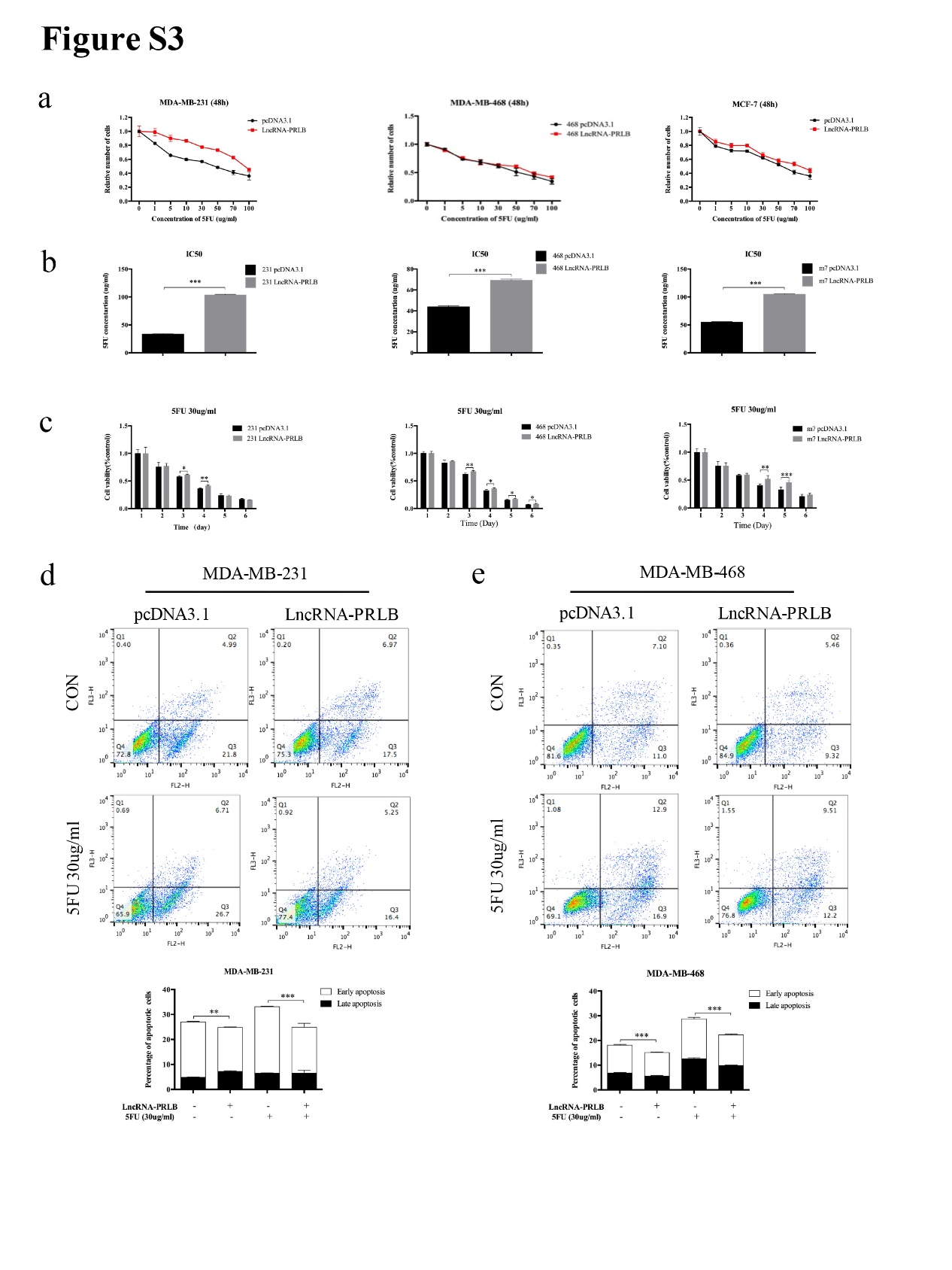


**Supplementary Figure S3** **LncRNA-PRLB overexpression inhibited the chemosensitivity of breast cancer cells to anticancer drugs.** (a) MTT assay performed in MDA-MB-231, MDA-MB-468 and MCF-7 cells transfected with pcDNA3.1-lncRNA-PRLB or pcDNA3.1 and treated with indicated concentrations of 5-FU. (b) The 5FU IC50 values of MDA-MB-231, MDA-MB-468 and MCF-7 cells was calculated from three independent experiments. (c) 30ug/ml 5FU was further used to test the inhibition of drug-resistance caused by lncRNA-PRLB overexpression in MDA-MB-231, MDA-MB-468 and MCF-7 cells. Flow cytometry of MDA-MB-231(d) and MDA-MB-468 (e) cells transfected with pcDNA3.1-lncRNA-PRLB or pcDNA3.1, followed by 5FU treatment. Representative results are shown, and data are presented as mean ± SD. (*P<0.05, **P<0.01, ***P<0.001, Student’s t test)


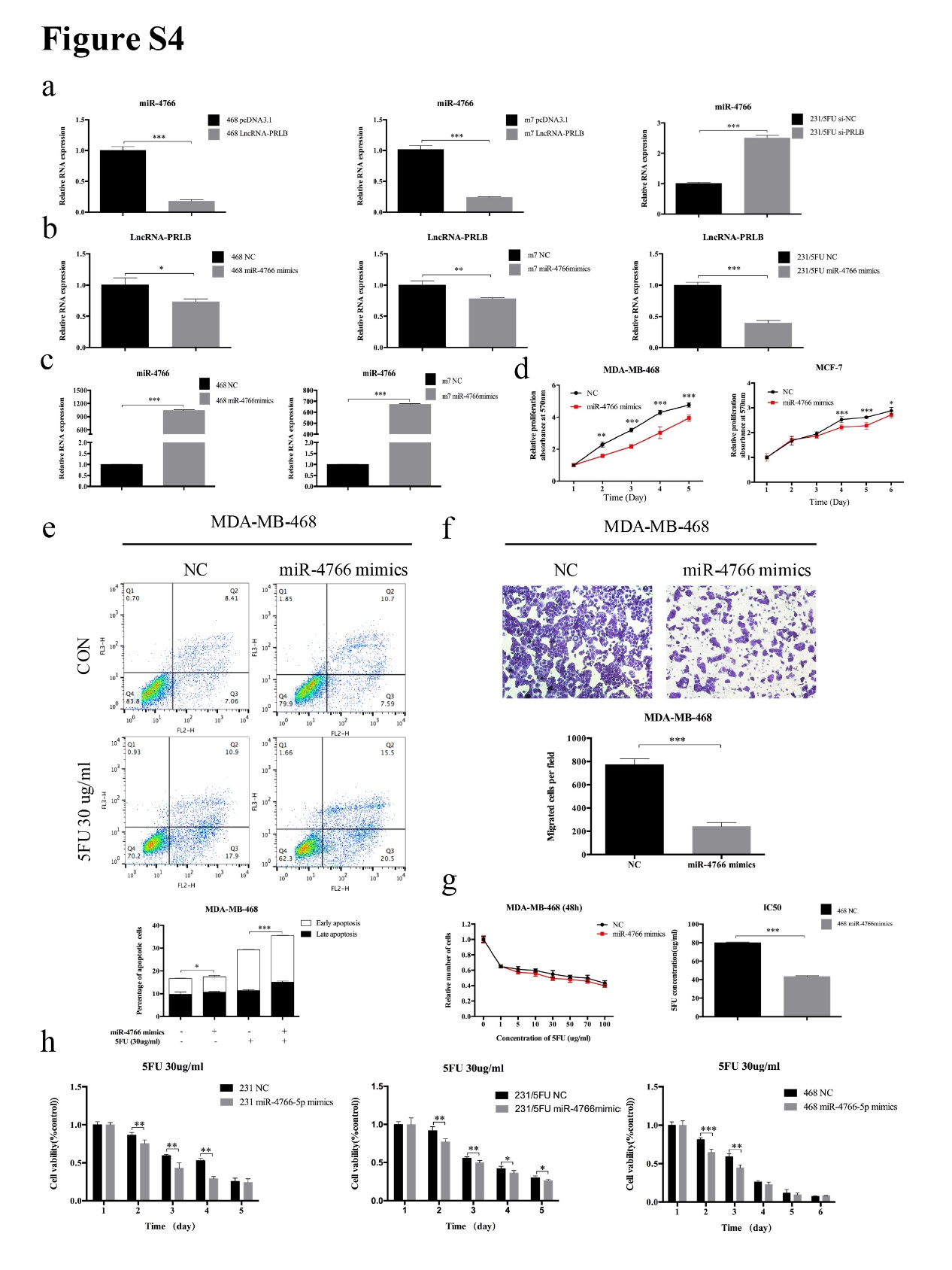


**Supplementary Figure S4 LncRNA-PRLB and miR-4766-5p could mutually regulate each other and miR-4766-5p overexpression inhibited cell proliferation, metastasis and chemoresistance in vitro.** (a) qRT-PCR was used to validate the changes of miR-4766 after lncRNA-PRLB overexpression in MDA-MB-468 and MCF7 cells or lncRNA-PRLB knockdown in MDA-MB-231/5FU cells. (b) LncRNA-PRLB expression was analyzed in MDA-MB-468, MCF-7 and MDA-MB-231/5FU cells transfected with miR-4766-5p mimics or NC. (c) The overexpression of miR-4766-5p in MDA-MB-468 and MCF7 cells was validated with qPCR. (d) MTT assays were used to determine the cell viability for miR-4766-5p overexpression in MDA-MB-468 and MCF-7 cells. (e) Flow cytometry of MDA-MB-468 cells transfected with miR-4766-5p mimics or NC, followed by 5FU treatment. (f) Transwell migration assays demonstrated that miR-4766-5p overexpression inhibited cell migration abilities. Columns are the average of three independent experiments. (g) MTT cell proliferation assay performed in MDA-MB-468 cells transfected with miR-4766-5p or NC and treated with the indicated concentrations of 5FU. Columns are the average of three independent experiments. (h) 30ug/ml 5FU was further used to test the inhibition of drug-resistance caused by miR-4766-5p overexpression in MDA-MB-231, MDA-MB-231/5FU and MDA-MB-468 cells. (**P*<0.05, **P<0.01, ***P<0.001, Student’s t test)


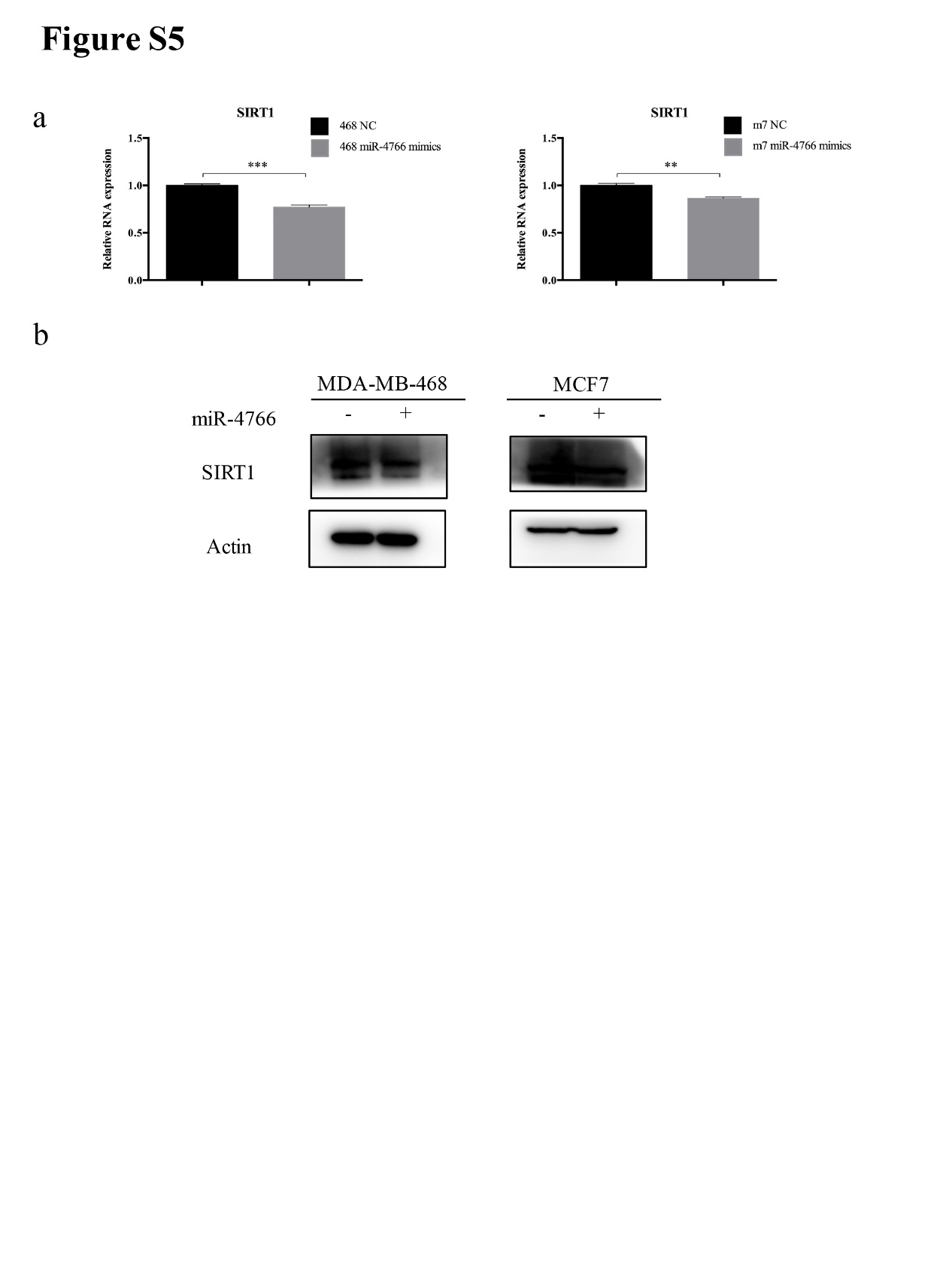


**Supplementary Figure S5 MiR-4766-5p suppressed SIRT1 expression both in mRNA and in protein levels.** (a) qPCR and (b) western blot analysis demonstrated that miR-4766-5p overexpression significantly reduced SIRT1 expression in MDA-MB-468 and MCF7 cells. (**P*<0.05, **P<0.01, ***P<0.001, Student’s t test)


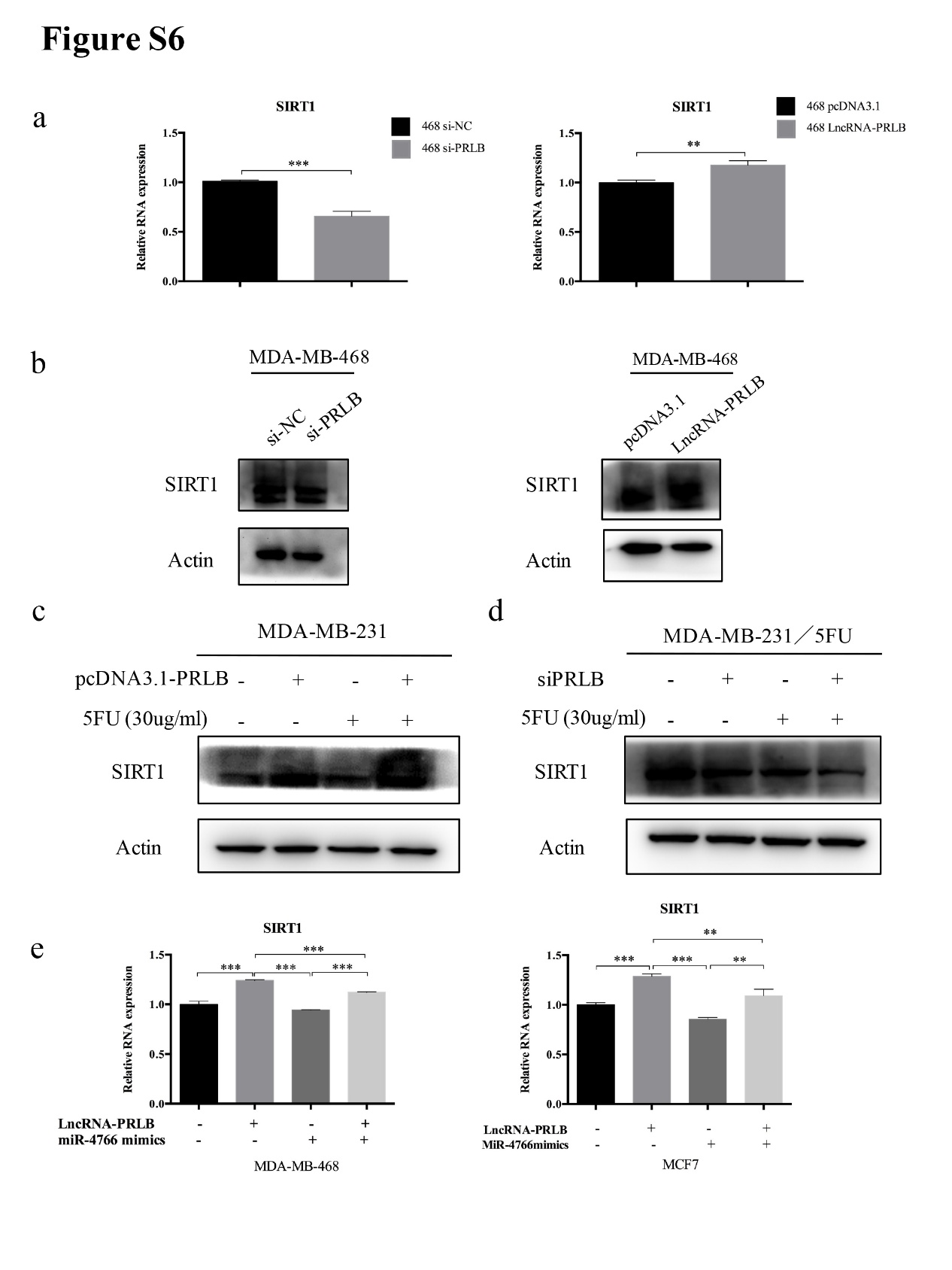


**Supplementary Figure S6 LncRNA-PRLB regulated SIRT1 expression partly through protecting it from miR-4766-5p-mediated degradation** (a) qPCR and (b) western blot analysis revealed lncRNA-PRLB knockdown or overexpression significantly influenced SIRT1 expression in MDA-MB-468 cells. (c) Western blot was used to analyze SIRT1 expression in MDA-MB-231 cells transfected with pcDNA3.1-PRLB or control, simultaneously treated with or without 5FU. (d) Western blot was used to analyze SIRT1 expression in MDA-MB-231/5FU cells transfected with si-PRLB or si-NC, simultaneously treated with or without 5FU. (e) SIRT1 expression was analyzed by qPCR in MDA-MB-468 or MCF7 cells transfected with pcDNA3.1-PRLB or control simultaneously with mimics of miR-4766-5p or NC. (*P<0.05, **P<0.01, ***P<0.001, Student’s t test)


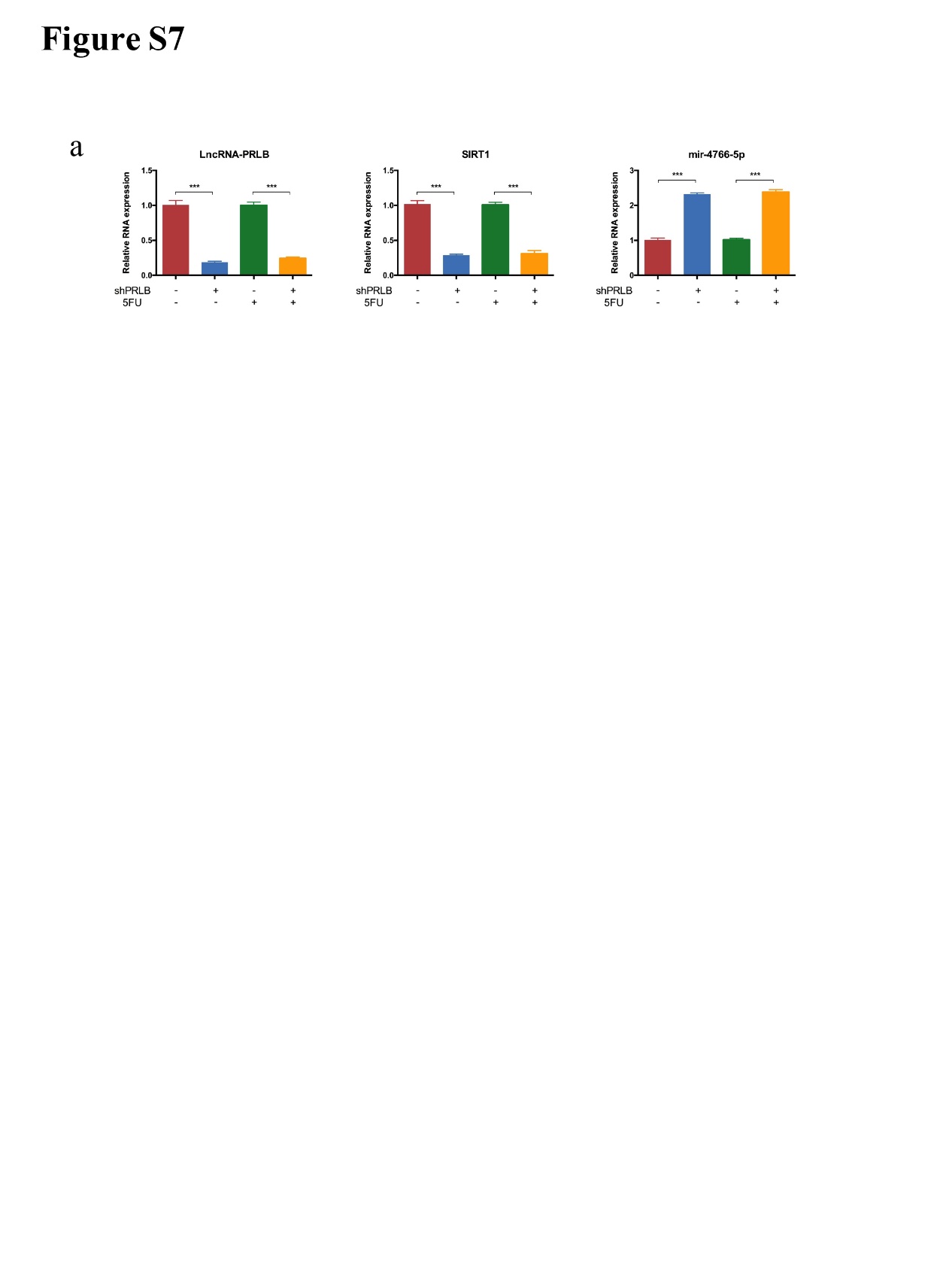


**Supplementary Figure S7 LncRNA-PRLB promoted SIRT1 expression and inhibited miR-4766-5p expression in vivo.** (a) lncRNA-PRLB and SIRT1 expression were lower in tumor tissues derived from shPRLB group than those from controls. MiR-4766-5p expression in shPRLB group was higher in tumor tissues derived from shPRLB group than those from controls. (*P<0.05, **P<0.01, ***P<0.001, Student’s t test)
