# Supplementary material for: A novel long non-coding RNA-PRLB acts as a tumor promoter through regulating miR-4766-5p/SIRT1 axis in breast cancer
Source: Cell Death Dis. 2018 May 11;9(5):563. doi: 10.1038/s41419-018-0582-1 (PMC5948209; doi:10.1038/s41419-018-0582-1)
Supplement: Supplementary file 3 — Supplementary Table II III IV V [file 41419_2018_582_MOESM3_ESM.docx]

| **Variables** | **Cases (%)** | **LncRNA-PRLB expression** | | ***P*-value^a^** |
| --- | --- | --- | --- | --- |
|  |  | **Low (n=33)** | **High (n=35)** |  |
| Age | | | | |
| ≤ 50 | 38 (55.9%) | 17 | 21 | 0.481 |
| > 50 | 30 (44.1%) | 16 | 14 |  |
| Tumor size (cm) | | | | |
| ≤ 2 | 22 (32.3%) | 11 | 11 | 0.867 |
| > 2 | 46 (67.6%) | 22 | 24 |  |
| Positive lymph nodes | | | | |
| 0 | 34 (50%) | 15 | 19 | 0.467 |
| ≥ 1 | 34 (50%) | 18 | 16 |  |
| Distant metastasis | | | | |
| M0 | 55 (80.9%) | 30 | 25 | 0.041 |
| M1 | 13 (19.1%) | 3 | 10 |  |
| Clinical stage | | | | |
| I | 15 (22.1%) | 5 | 10 | 0.203 |
| II | 32 (47.1%) | 19 | 13 |  |
| III | 8 (11.8%) | 6 | 2 |  |
| IV | 13 (19.1%) | 3 | 10 |  |
| ER | | | | |
| Negative | 11 (16.2%) | 7 | 4 | 0.274 |
| Positive | 57 (83.8%) | 26 | 31 |  |
| PR | | | | |
| Negative | 14 (20.6%) | 9 | 5 | 0.185 |
| Positive | 54 (79.4%) | 24 | 30 |  |
| HER-2 | | | | |
| Negative | 65 (95.6%) | 32 | 33 | 0.59 |
| Positive | 3 (4.4%) | 1 | 2 |  |

Supplementary Table II. Associations between patient characteristics and LncRNA-PRLB expression

^a^Chi-square detection.

Supplementary Table III. Influence of lncRNA-PRLB expression and different clinicopathological parameters on overall survival for breast cancer patients

| **Univariate analysis** | ***P*-value^a^** |
| --- | --- |
| Age | 0.231 |
| Tumor size | 0.271 |
| Positive lymph nodes | 0.031 |
| Distant metastasis | NA^b^ |
| Clinical stage | NA^b^ |
| LncRNA-PRLB expression | 0.024 |

^a^Kaplan-Meier survival analysis.

^b^Data are not available due to low number of patients.

Supplementary Table IV. Cox proportional hazard multivariate analysis: influence of lncRNA-PRLB levels and positive lymph nodes on overall survival for breast cancer patients

| **Multivariate analysis** | ***P*-value^a^** | **Hazard ratio** | **Confidence interval** | |
| --- | --- | --- | --- | --- |
| Age | 0.098 | 0.282 | 0.063 | 1.262 |
| Tumor size | 0.506 | 1.615 | 0.394 | 6.627 |
| Positive lymph nodes | 0.075 | 0.130 | 0.014 | 1.230 |
| LncRNA-PRLB expression | 0.046 | 0.112 | 0.013 | 0.965 |

^a^Cox proportional hazards model multivariate analysis.

Supplementary Table V. Primers used for qRT-PCR.

| **Gene** | **Forward** | **Reverse** |
| --- | --- | --- |
| LncRNA-PRLB | CAGAGTTGACCCTGGGCTTTC | CATCCTTGCCTCCACTCCTCA |
| ENST00000438893 | CAGACATACTTTATCATCCCTT | ACAATGCCACCTCCTCC |
| ENST00000410119 | CTGCTTGCCTCGGCAG | CTTTTAATGGAACACTTCATG |
| ENST00000452199 | CAAAGGCTAGACGGGAGC | CATGGCAAGGAGGGAACT |
| ENST00000514980 | GCAACCTCCAACTCCCTG | GGCTCGCACCTGTAATCC |
| SIRT1 | TAGACACGCTGGAACAGGTTGC | CTCCTCGTACAGCTTCACAGTC |
| Actin | CATGTACGTTGCTATCCAGGC | CTCCTTAATGTCACGCACGAT |
